# Supplementary material for: A pocket-escaping design to prevent the common interference with near-infrared fluorescent probes in vivo
Source: Nat Commun. 2020 Mar 26;11:1573. doi: 10.1038/s41467-020-15323-8 (PMC7099068; doi:10.1038/s41467-020-15323-8)
Supplement: Supplementary file 1 — Supplementary Information [file 41467_2020_15323_MOESM1_ESM.pdf]

Supplementary Information

A pocket-escaping design to prevent the common interference with near-infrared fluorescent probes *in vivo*

Xing et al.

Supplementary Methods

Experimental materials

All the chemical reagents were commercially provided, of analytical grade and used without further purification. ^{1}H NMR and ^{13}C NMR spectra were recorded on Bruker 600 MHz AVANCE III spectrometer, BRUKER Ltd. Mass spectra were obtained with Thermo Scientific MSQ Plus mass spectrometer (USA). Absorption spectra were collected by using HACH DR6000 UV/VIS Spectrophotometer (USA). Viscosity experiments were carried out using an Ubbelohde viscometer. The fluorescence imaging of probe distribution in mice and probe electrophoretic bands in native PAGE gels were respectively performed using an IVIS Lumina XR (XENOGEN, Caliper, Hopkinton, MA, USA) imaging system. Column chromatography was carried out on silica gel (G200−300 mesh, Qingdao Haiyang Chemical Co., Qingdao, China). Fluorescent spectra of time-course and titration experiments were recorded on FluoroMax-4 spectrofluorometer (Horiba Jobin Yvon Inc.). The other spectra were measured with a Thermo Scientific Lumina Fluorescence Spectrometer (USA). Quantitative measurements of probes binding to albumin were conducted on a Malvern Microcal PEAQ ITC. Haematoxylin and eosin (H&E) staining images were captured using Axio Imager A2 microscope. Masson staining pictures were captured using an inverted microscope (IX73, Olympus). Molecular docking of all compounds was performed via Discovery Studio (BIOVIA, USA). Fluorescent probe pharmacokinetics and biodistribution were detected with FlexStation III Multi-Mode Microplate Reader (Molecular Devices, USA).

The three-dimensional structures of TICT dyes were constructed using Chem. 3D ultra 14.0 software (Chemical Structure Drawing Standard; Cambridge Soft Corporation, USA). Fluorescent spectra data were plotted using Origin (Ver. 9.5) graphing software. Heatmaps and histograms were drawn in GraphPad Prism software (Ver. 7.02). Molecular docking of all compounds was performed via Discovery Studio Ver. 3.5 (BIOVIA, USA) as implemented through the graphical user interface CDOCKER protocol. DFT calculations

were carried out using the Gaussian 09 program package. NMR results were analyzed using MestReNova Ver. 9.0.1 (Mnova). ITC results were processed by the build-in software of Microcal PEAQ ITC analysis.

In vivo Pharmacokinetics and Biodistribution study

For pharmacokinetic analysis, the probes were administered intravenously to C57BL/6 female mice at a dose of 200 μM, 100 μL for each and blood was withdrawn from retro-orbital plexus of mice at 3, 5, 10, 30, 60, 120, and 240 min post-injection using heparinised microcentrifuge tubes. Plasma was separated by centrifuging the blood samples at 10,000 rpm for 10 min. 50 μL plasma was added to 450 μL methanol in micro 1.5-mL plastic centrifuge tubes. The tubes were vortex-mixed for 30 s and centrifuged at 10,000 rpm for 10 min.

For the biodistribution study of probes, equivalent doses of BNLBN and NLBN (200 μM, 100 μL for each) were intravenously injected and the mice were sacrificed at 1, 3, and 6 h. The heart, liver, spleen, lung, kidney, and intestine tissues were taken. The surface blood was washed with physiological saline and dried with filter paper. After weighing all of the tissues, they were homogenated with methanol (m:V = 1:2, m is the mass of tissue and V is the volume of methanol), the tissue homogenate was centrifuged at 10,000 rpm for 10 min, and the supernatant was extracted.

Deproteinized plasma and tissue supernatant were analyzed on a 96-well microplate at volumes of 150 μL and fluorescence detection was performed by a microplate reader (Molecular Devices, USA). At least three animals were analyzed at each time point. Results were presented as mean. The plasma concentrations vs time profiles were analyzed with DAS 3.0 software. A double-component model of pharmacokinetic was utilized to calculate the pharmacokinetic parameters.

In vivo Toxcicity of probes

To evaluate the toxicity of probes *in vivo*, C57BL/6 female mice were randomly divided into three groups, two of which were intravenously administered with NLBN and BNLBN (200 μM, 100 μL for each) every other day for 4 days, and another group injected with PBS was utilized as control. The weight and physical behavior of these groups of mice were monitored closely every two days for 14 days post injection. On the 14th day post probes injection, the mice were sacrificed. Blood samples were harvested to test the main hepatic indicators, alanine aminotransferase (ALT), aspartate aminotransferase (AST), blood urea nitrogen (BUN), and lactate dehydrogenase (LDH). After the blood collection, hearts, livers, spleens, lungs, and kidneys of animals from different groups were collected and fixed in 4% paraformaldehyde for H&E staining.

Electrophoresis

All the samples were analyzed with 8% native PAGE. The gels were observed by fluorescence under the IVIS Lumina XR (XENOGEN, Caliper, Hopkinton, MA, USA) and then stained with Coomassie brilliant blue. Additionally, the albumin binding property of dyes was further investigated by adding 10 μM NLB or BNLB to a solution of excess BSA (100 μM in PBS, 10 mM, pH 7.4). The dyes and BSA were incubated at 37 °C for 1 h, and the incubation samples were analyzed with 8.0% native PAGE.

Compound Synthesis

Characterization of the below compounds can be found in Supplementary Figs 23-42.

Synthesis of DCM

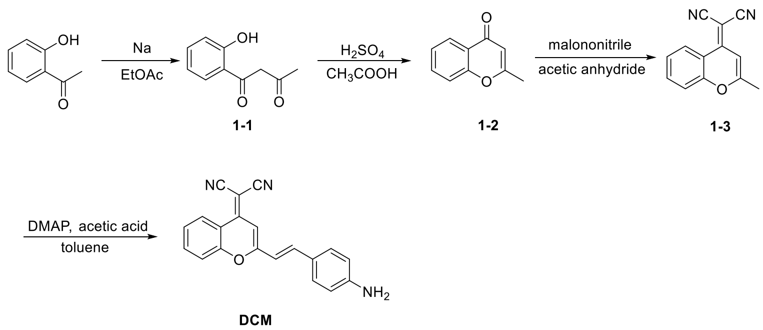

1-1 2'-Hydroxyacetophenone (2.26 g, 16.6 mmol) was dissolved in 50 mL ethyl acetate, and then sodium (1.5 g, 65.0 mmol) was added into the solution. The mixture was violently stirred for 4 h at room temperature. Then 4 M hydrochloric acid solution was added dropwise to adjust its pH to neutral. A grayish-green solid was filtered and directly used in the next reaction without further purification (61%).

1-2 Concentrated sulfuric acid (2 mL) was slowly added to acetic acid solution (30 mL) containing compound **1-1** (1.78 g, 10.0 mmol). The mixture was refluxed for about 1 h and then was poured into 200 mL ice water, followed by adjusting its pH to neutral with Na_2CO_3. The aqueous solution was extracted with dichloromethane twice. The organic layers were combined and dried over Na_2SO_4. The crude mixture was purified on silica gel chromatography (DCM/PE = 1/1, v/v) to obtain the product as a colorless solid (51%). ^{1}H NMR (600 MHz, $CDCl_3$) δ 8.22 – 8.11 (m, 1H), 7.73 – 7.53 (m, 1H), 7.38 – 7.28 (m, 1H), 6.25 – 6.07 (m, 1H), 2.36 (t, 3H).

1-3 Compound **1-2** (800 mg, 5.0 mmol) and malononitrile (495 mg, 7.5 mmol) were dissolved in 25 mL of acetic anhydride. The solution was refluxed for 14 h and then the solvent was evaporated in vacuum. The crude product was purified by silica gel chromatography to yield compound **1-3** as an orange solid (33% yield). ^{1}H NMR (600 MHz, $CDCl_3$) δ 8.91 (d, *J* = 8.2 Hz, 1H), 7.81 – 7.68 (m, 1H), 7.45 (t, *J* = 8.2 Hz, 2H), 6.72 (s, 1H), 2.44 (s, 3H).

DCM Compound **1-3** (208 mg, 1.0 mmol) and 4-acetamidobenzaldehyde (245 mg, 1.5 mmol) were dissolved in toluene (20 mL) with DMAP (125 mg, 1.0 mmol) and acetic acid (0.1 mL) under N_2 atmosphere. Then the mixture was refluxed for 3 h and the solvent was evaporated under reduced pressure. The product was refluxed in a solution of concentrated hydrochloric acid and ethanol (2/1, v/v, 10 mL) for another 2 h before the pH

of the solution was adjusted to neutral. The mixture was concentrated to obtain the crude product, which was purified by silica gel chromatography to yield the product as a crimson solid (38% yield). Characterization data are in agreement with literature report.[1] ^{1}H NMR (600 MHz, DMSO-d_6) δ 10.56 (s, 2H), 9.78 (s, 2H), 7.76 (t, 2H), 7.75 (t, 2H), 6.94 (t, 2H), 6.92 (t, 2H), 6.86 (d, J = 8.6 Hz, 1H).

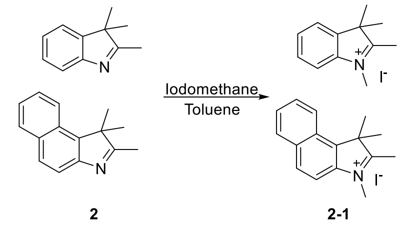

Iodine salt

Corresponding compound (10.0 mmol) and iodomethane (940 μL, 15.0 mmol) were dissolved in toluene (20 mL) and the mixture refluxed for 12 h. Then the product was filtered and washed with diethyl ether to obtain the final product without further purification and used directly in the next.

Cy7

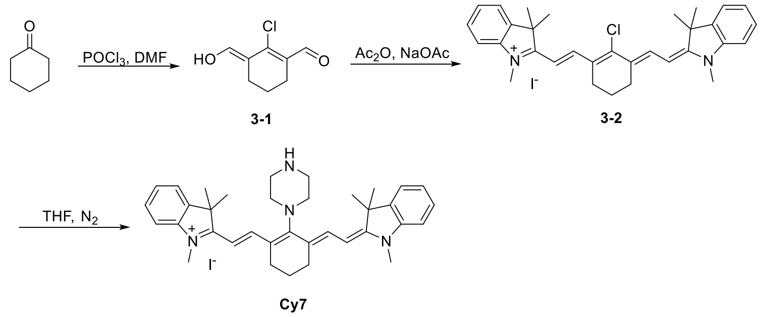

3-1 $POCl_3$ (4 mL) was added dropwise to a solution of DMF (20 mL) in an ice bath. And the solution was stirred for 30 min under N_2 atmosphere. Cyclohexanone (2 mL) in 5 mL DMF was added dropwise to the mixture. Then the solution was heated to 60 °C for another 5 h. Cooled and poured it into ice water (200 mL) to give a yellow solid, which was used directly without further purification.

3-2 **3-1** (200 mg, 1.2 mmol), indole iodine salt (687 mg, 2.4 mmol) and sodium acetate (197 mg, 2.4 mmol) were dissolved in Ac_2O (20 mL). The mixture was heated to 70 °C for 3 h. Then the solvent was removed under reduced pressure. The crude product was

washed with diethyl ether and water to give the product as a green solid (73% yield). Characterization data are in agreement with literature report.[2] ^{1}H NMR (600 MHz, DMSO-*d*$_6$) δ 8.24 (d, *J* = 7.4 Hz, 2H), 7.63 (d, *J* = 7.4 Hz, 2H), 7.52 – 7.37 (m, 4H), 7.34 – 7.18 (m, 2H), 6.30 (d, *J* = 7.2 Hz, 2H), 3.69 (s, 6H), 2.72 (t, *J* = 7.8 Hz, 4H), 1.89 – 1.82 (m, 2H), 1.67 (s, 12H).

Cy7 **3-2** (319 mg, 0.5 mmol) and piperazine (172 mg, 2 mmol) were dissolved in anhydrous THF (10 mL) and the mixture was refluxed for 4 h under a N_2 atmosphere. Then the solvent was removed under reduced pressure. The crude product was purified by silica gel chromatography using ethanol/DCM (1/20, v/v) as eluent to give a golden powder (58% yield). Characterization data are in agreement with literature report.[3] ^{1}H NMR (600 MHz, DMSO-*d*$_6$) δ 7.59 (d, *J* = 7.5 Hz, 1H), 7.51 (d, *J* = 6.9 Hz, 1H), 7.34 (m, *J* = 7.9 Hz, 1H), 7.24 (d, *J* = 7.9 Hz, 1H), 7.12 (m, *J* = 7.5 Hz, 1H), 5.88 (d, *J* = 7.5 Hz, 1H), 3.74 – 3.67 (m, 2H), 3.51 (s, 4H), 3.34 (s, 3H), 3.10 – 3.03 (m, 2H), 1.79 – 1.70 (m, 1H), 1.62 (s, 7H).

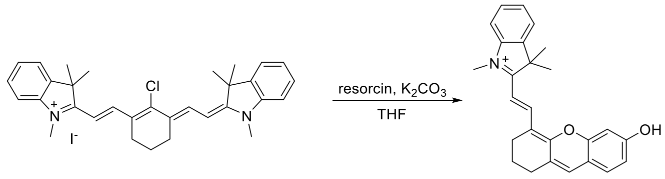

3-2 H-Cy7

H-Cy7 A solution of resorcin (110 mg, 1.0 mmol) in dry THF (20 mL) was added K_2CO_3 (276 mg, 2.0 mmol) and the resulting mixture was stirred at room temperature for 30 min under N_2 atmosphere. Then a solution of **3-2** (319 mg, 0.5 mmol) in THF (5 mL) was added to the above mixture via syringe, and the reaction was heated to 50 °C for 5 h. The solvent was evaporated under reduced pressure. The crude product was purified by silica gel chromatography using DCM/MeOH as eluent to afford the product as a blue-green solid (54% yield). Characterization data are in agreement with literature report.[4] ^{1}H NMR (600 MHz, DMSO-*d*$_6$) δ 8.33 (d, *J* = 14.4 Hz, 1H), 7.62 (d, *J* = 7.4 Hz, 1H), 7.55 (s, 1H), 7.47 (s, 1H), 7.46 – 7.40 (m, 4H), 7.32 – 7.25 (m, 1H), 6.71 (dd, *J* = 8.7, 2.0 Hz, 1H), 6.66 (s, 1H), 6.22 (d, *J* = 14.4 Hz, 1H), 3.68 (s, 5H), 2.68 (t, 3H), 2.64 (t, *J* = 5.9 Hz, 3H), 1.84 – 1.78 (m, 3H), 1.70 (s, 8H).

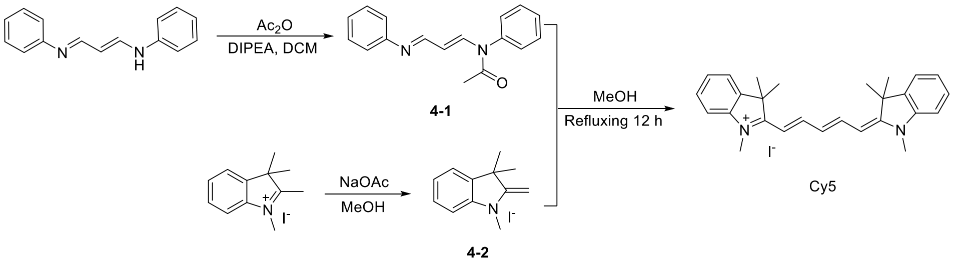

Cy5 A solution of acetic anhydride (50 μL) in DCM (0.5 mL) was added dropwise to a mixture of 3-anilinoacrolein anil (222 mg, 1.0 mmol) and DIPEA (100 μL) in DCM (2 mL) at 0 °C. The mixture was reacted at room temperature with stirring for 3 h. The solvent was evaporated under reduced pressure to obtain **4-1** and used directly without further purification. Indole iodine salt (600 mg, 2.0 mmol) and sodium acetate (164 mg, 2.0 mmol) were dissolved in MeOH (5 mL). The mixture was stirred for 3 h at room temperature. Then added to the flask of **4-1**. The solution was refluxed for 12 h. The solvent was evaporated, and the precipitate was washed with diethyl ether three times to give a green solid (78% yield). Characterization data are in agreement with literature report.[5] ^{1}H NMR (600 MHz, DMSO-d_6) δ 8.32 (t, 2H), 7.61 (d, J = 7.3 Hz, 2H), 7.42 – 7.37 (m, 4H), 7.28 – 7.17 (m, 2H), 6.55 (s, 1H), 6.27 (d, J = 7.9 Hz, 2H), 1.67 (s, 12H), 1.26 (t, J = 7.0 Hz, 6H).

Synthesis of NC

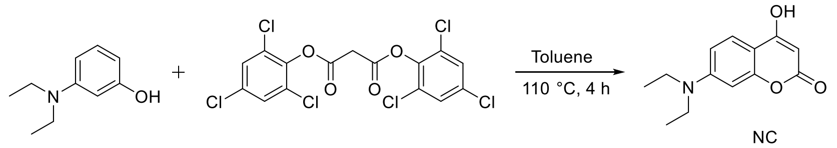

Bis(2,4,6-trichlorophenyl) malonate (462 mg, 1.0 mmol) and 3-diehylaminopheniol (1.0 mmol) were dissolved in toluene and the resulting reaction was heated at reflux for 4 h. The desired product was precipitated and the solvent was evaporated. The final product was purified by silica gel chromatography using DCM/PE as eluent to afford the product (63% yield). Characterization data are in agreement with literature report.[6] ^{1}H NMR (600 MHz, DMSO-d_6) δ 11.89 (s, 1H), 7.54 (d, J = 8.7 Hz, 1H), 6.65 (d, J = 8.3 Hz, 1H), 6.44 (s, 1H), 5.25 (s, 1H), 3.40 (d, J = 8.6 Hz, 4H), 1.11 (t, J = 8.2 Hz, 6H).

Synthesis of BNC

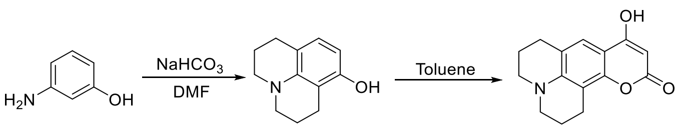

5 BNC

Synthesis of 8-hydroxyjulolidine (**5**)

To a solution of 3-aminophenol (437 mg, 4.0 mmol) in DMF added 1-bromo-3-chloropropane (2.52 g, 16.0 mmol) and sodium bicarbonate (1.34 g, 16.0 mmol). The reaction mixture was stirred at 70 °C for 10 h. Then the reaction mixture was extracted with EtOAc (150 mL), washed with water (3 × 150 mL) and dried over $MgSO_4$. The solvent was evaporated and the product was purified by silica gel chromatography using DCM/PE (3/1, v/v) to give a white product (45%).

Bis(2,4,6-trichlorophenyl) malonate (462 mg, 1.0 mmol) and 8-hydroxyjulolidine (1.0 mmol) were dissolved in toluene and the resulting reaction was heated at reflux for 4 h. Then the solvent was evaporated. The final product was purified by silica gel chromatography using DCM/PE as eluent to afford the product (48% yield). Characterization data are in agreement with literature report.[7] ^{1}H NMR (600 MHz, $CDCl_3$) δ 7.48 (s, 1H), 5.45 (s, 1H), 3.35 (dd, *J* = 6.7 Hz, 4H), 2.87 (t, *J* = 6.4 Hz, 2H), 2.83 – 2.72 (m, 2H), 2.03 – 1.86 (m, 4H).

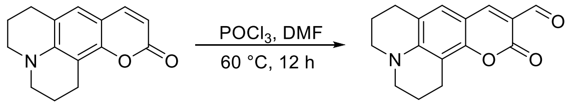

BNCQ

$POCl_3$ (2.0 mL) was added dropwise to a DMF (5.0 mL). The solution was stirred at 0 °C under N_2 atmosphere for 30 min to obtain a canary colored solution. A solution of coumarin 6H (965 mg, 4.0 mmol) was then added to the above solution. The mixed solution was first stirred at room temperature for 30 min, then at 60 °C for another 12 h. The reaction mixture was added into ice water (100 mL) and aged for 2 h. NaOH solution (20%) was added to adjust the solution's pH to 7.0 to yield a precipitate. Filtered to afford the final product. Characterization data are in agreement with literature report.[8] ^{1}H NMR (600 MHz, DMSO-*d*$_6$) δ 9.86 (d, *J* = 4.2 Hz, 1H), 8.21 (dd, *J* = 4.2, 1.7 Hz, 1H), 7.23 (d, *J* = 4.1 Hz, 1H), 3.37 (dd, *J* = 4.3 Hz, 4H), 2.86 – 2.57 (m, 4H), 1.98 – 1.78 (m, 4H).

Synthesis of BNCN

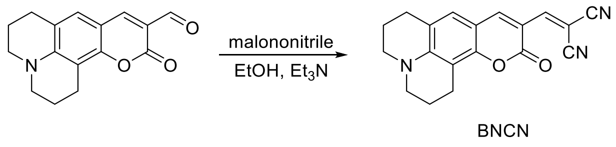

Under an atmosphere of N_2, BNCQ (1.0 mmol) and malononitrile (132 mg, 2.0 mmol) was stirred at room temperature in absolute EtOH (10 mL) overnight with trace Et_3N as catalyst. Then, the resultant was filtered and washed with EtOH to obtain a blue-purple solid. The final product was purified by silica gel chromatography using DCM/MeOH as eluent to afford the product BNCN (45%). Characterization data are in agreement with literature report.[9] ^{1}H NMR (600 MHz, DMSO-d_6) δ 8.46 (s, 1H), 7.92 (s, 1H), 7.22 (s, 1H), 3.45 (dd, J = 7.3 Hz, 4H), 2.72 (dd, J = 7.9 Hz, 4H), 1.97 – 1.82 (m, 4H). ^{13}C NMR (150 MHz, DMSO-d_6) δ 159.81, 153.30, 153.14, 151.15, 144.65, 129.04, 121.56, 116.27, 115.01, 108.89, 107.99, 106.14, 72.97, 50.75, 50.15, 26.88, 20.72, 19.70.

Synthesis of BNCY and BNCE

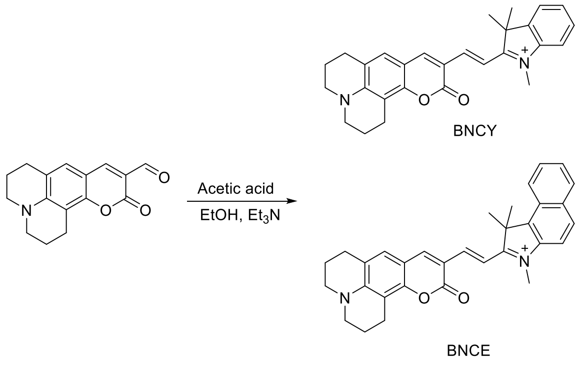

BNCQ (1.0 mmol) and corresponding indole salt (2.0 mmol) was refluxing in absolute EtOH (10 mL) for 10 h with trace Et_3N and acetic acid as catalyst. Then the solvent was evaporated under reduced pressure. The final product was purified by silica gel chromatography using DCM/MeOH as eluent to afford the product BNCY and BNCE (55% and 41%, respectively). BNCY, ^{1}H NMR (600 MHz, DMSO-d_6) δ 8.66 (s, 1H), 8.25 (d, J = 8.7 Hz, 1H), 7.80 (t, J = 8.2 Hz, 2H), 7.74 (d, J = 8.7 Hz, 1H), 7.58 (t, J = 7.6 Hz, 1H), 7.53 (t, J = 7.4 Hz, 1H), 7.17 (s, 1H), 3.93 (s, 3H), 3.46 (d, J = 5.4 Hz, 4H), 2.77 (t, J = 5.8 Hz, 4H), 1.91 (s, 4H), 1.75 (s, 6H). ^{13}C NMR (151 MHz, DMSO-d_6) δ 180.73, 159.94, 152.79, 150.73, 150.06, 143.33, 142.45, 129.31, 128.64, 128.32, 123.18, 121.48, 114.61, 111.24,

109.97, 109.20, 105.90, 51.55, 50.71, 50.12, 33.82, 27.09, 26.57, 20.82, 19.87, 19.79. m/z: $[M]^+$ Calcd. for $C_{28}H_{29}N_2O_2^+$ 425.2224, Found 425.2229. BNCE, ^1H NMR (600 MHz, DMSO-d_6) δ 8.71 (s, 1H), 8.40 (d, J = 8.5 Hz, 1H), 8.34 (d, J = 8.8 Hz, 1H), 8.24 (d, J = 8.9 Hz, 1H), 8.18 (d, J = 8.2 Hz, 1H), 8.04 (d, J = 8.9 Hz, 1H), 7.82 – 7.75 (m, 2H), 7.70 – 7.65 (m, 1H), 7.18 (s, 1H), 4.07 (s, 3H), 3.45 (dd, J = 6.3 Hz, 4H), 2.77 (dd, J = 5.4 Hz, 4H), 1.98 (s, 6H), 1.91 (dd, J = 5.9 Hz, 4H). ^{13}C NMR (150 MHz, DMSO-d_6) δ 181.54, 160.08, 152.72, 150.55, 149.15, 148.78, 140.04, 137.36, 133.20, 131.19, 130.49, 128.73, 128.23, 127.30, 127.09, 123.43, 121.38, 113.40, 111.28, 109.93, 108.88, 105.88, 53.25, 50.68, 50.09, 34.40, 27.11, 26.39, 20.83, 19.88, 19.81. m/z: $[M]^+$ Calcd. for $C_{32}H_{31}N_2O_2^+$ 475.2380, Found 475.2388.

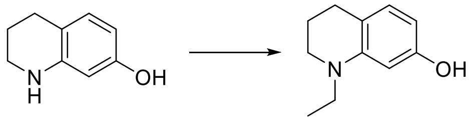

BOH

1-ethyl-1,2,3,4-tetrahydroquinolin-7-ol (BOH)

1,2,3,4-Tetrahydro-quinolin-7-ol (600 mg, 4.0 mmol) was suspended in water followed by the addition of sodium bicarbonate (380 mg, 4.5 mmol) and tetrabutylammonium bromide (TBAB, 32 mg, 0.1 mmol). Then bromoethane (500 µL, 6.7 mmol) was added dropwise to the mixture at 0 °C. The reaction mixture was brought to room temperature and left for 12 h. The aqueous reaction mixture was extracted with ethyl acetate, dried with sodium sulfate, and evaporated to dryness. The crude mixture was purified on silica gel (DCM/EtOAc = 20:1, v/v) to give a light brown solid with the yield of 55%. Characterization data are in agreement with literature report.[10] ^1H NMR (600 MHz, $CDCl_3$) δ 6.76 (d, J = 8.0 Hz, 1H), 6.10 (d, J = 8.1 Hz, 1H), 6.02 (dd, J = 7.9 Hz, 1H), 3.27 (q, J = 7.9 Hz, 2H), 3.22 (t, 2H), 2.65 (t, J = 8.4 Hz, 2H), 1.11 (t, J = 8.1 Hz, 3H).

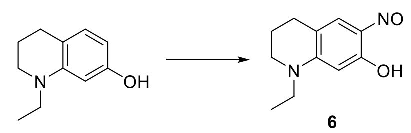

6

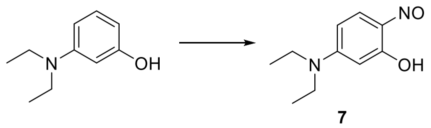

7

Synthesis of **6** and **7**

Corresponding aromatic phenolic compound (4.0 mmol) or was dissolved in a mixture of

ice water (15 mL) and concentrated hydrochloric acid (3 mL). The solution was cooled to 0 °C and a solution of sodium nitrite (290 mg, 4.2 mmol) in ice water (2 mL) was added dropwise over a period of 30 min and the stirring was continued at 0 °C for 2 h. The solution was filtered to give a brown solid and this compound was used in the next without further purification.

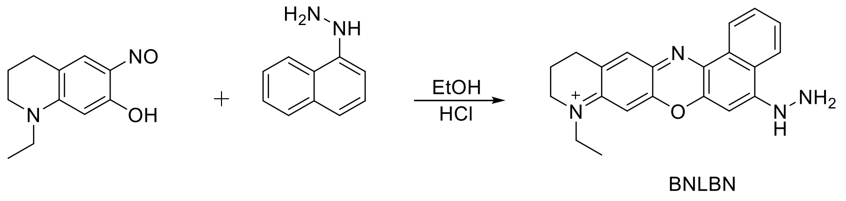

Synthesis of BNLBN

The nitroso compound **6** (206 mg, 1.0 mmol) and 1-Naphthylhydrazine hydrochloride (195 mg, 1.0 mmol) was dissolved in ethanol (20 mL), cooled to 0 °C in an ice bath, then treated with concentrated HCl (2 mL), stirred at 0 °C for 1 h. Then the mixture was refluxed for another 10 h. Concentrated the mixture in vacuo to a deep red-purple residue. The crude product was purified on silica gel (DCM/MeOH = 20:1, v/v) to afford a violet gold solid with a yield of 18%. ^{1}H NMR (600 MHz, DMSO-d_6) δ 8.70 (dd, *J* = 8.2 Hz, 1H), 8.38 (d, *J* = 8.1 Hz, 1H), 8.17 (s, 2H), 7.94 – 7.87 (m, 1H), 7.83 – 7.76 (m, 1H), 7.53 (s, 1H), 6.75 (s, 1H), 6.60 (s, 1H), 3.49 – 3.44 (m, 2H), 3.10 (q, *J* = 8.3 Hz, 2H), 2.86 (d, *J* = 8.0 Hz, 2H), 1.89 (dd, *J* = 8.2 Hz, 2H), 1.17 (t, *J* = 8.3 Hz, 3H). ^{13}C NMR (150 MHz, DMSO-d_6) δ 159.81, 154.69, 150.79, 147.31, 132.36, 131.79, 131.70, 131.42, 131.12, 129.38, 126.30, 124.29, 124.02, 122.67, 96.83, 96.34, 46.25, 44.68, 41.63, 40.50, 26.58, 20.14, 9.11. m/z: [M]$^+$ Calcd. for $C_{32}H_{31}N_2O_2^+$ 475.2380, Found 475.2388. m/z: [M]$^+$ Calcd. for $C_{21}H_{21}N_4O^+$ 345.1710, Found 345.1719.

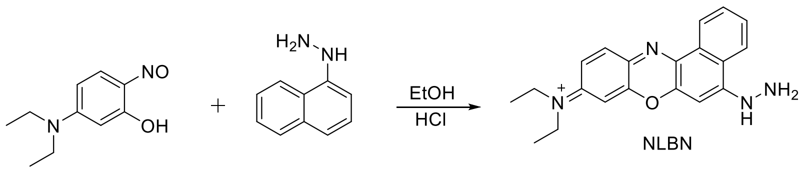

Synthesis of NLBN

The nitroso compound **7** (194 mg, 1.0 mmol) and 1-Naphthylhydrazine hydrochloride (195 mg, 1.0 mmol) was dissolved in ethanol (20 mL), cooled to 0 °C in an ice bath, then treated with concentrated HCl (2 mL), stirred at 0 °C for 1 h. Then the mixture was refluxed for

another 10 h. Concentrated the mixture in vacuo to a deep red-purple residue. The crude product was purified on silica gel (DCM/MeOH = 20:1, v/v) to afford a violet gold solid with a yield of 34%. ^{1}H NMR (600 MHz, DMSO-d_6) δ 7.48 (dd, J = 9.1 Hz, 1H), 6.83 – 6.79 (m, 1H), 6.64 (d, J = 8.6 Hz, 1H), 6.37 – 6.34 (m, 1H), 6.23 (d, J = 9.4 Hz, 1H), 6.19 (s, 2H), 3.45 (q, J = 9.0 Hz, 4H), 3.33 (s, 1H), 1.13 (t, J = 9.0 Hz, 6H). ^{13}C NMR (150 MHz, DMSO-d_6) δ 179.48, 149.17, 149.00, 145.80, 145.08, 142.50, 129.75, 126.11, 111.02, 103.12, 100.24, 96.28, 44.72, 12.90. m/z: $[M]^+$ Calcd. for $C_{20}H_{21}N_4O^+$ 333.1710, Found 333.1718.

Supplemental Figures

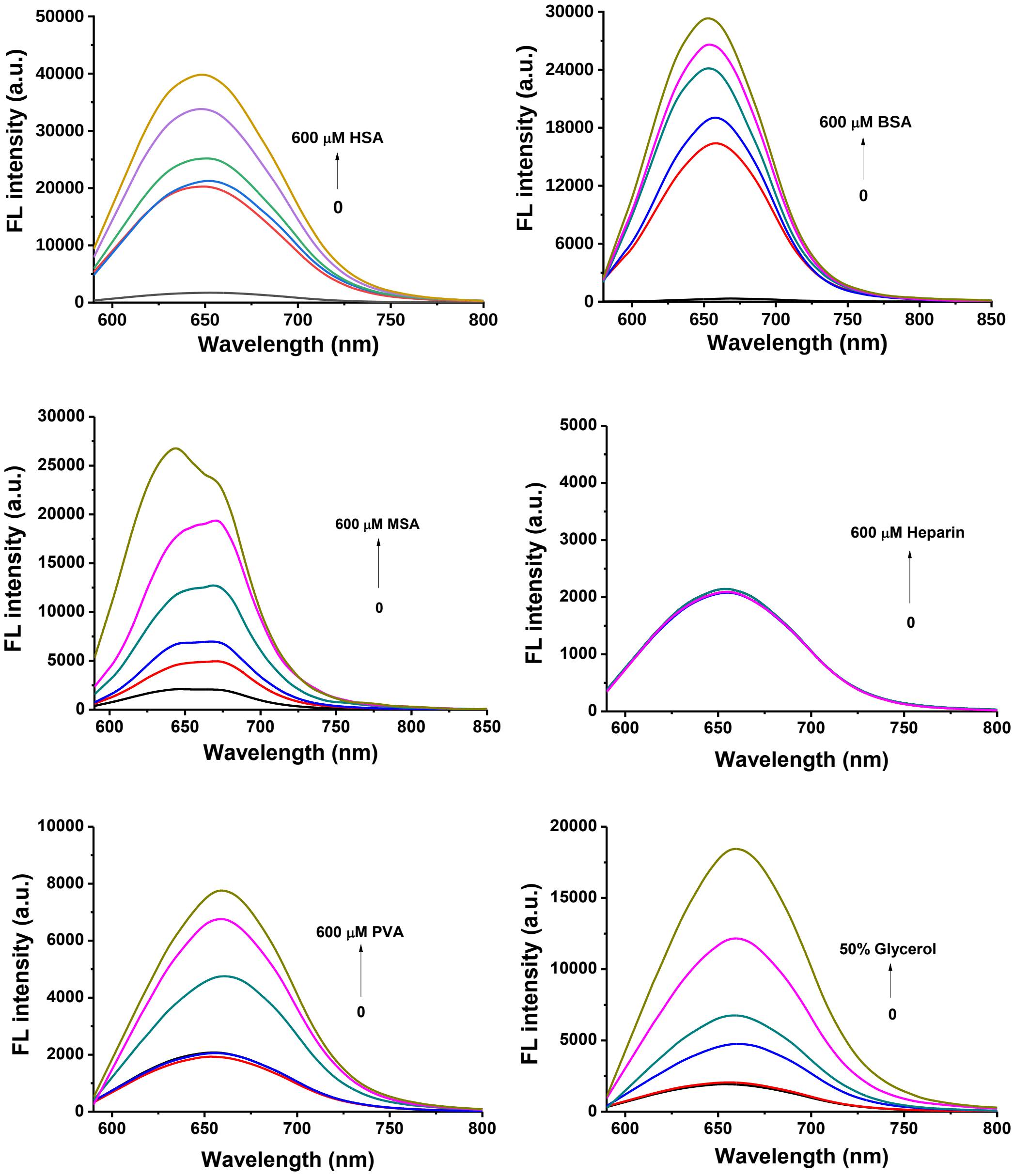

Supplementary Figure 1. Fluorescence examination of DCM. Fluorescence intensity changes of DCM (10 μM) upon addition of HSA, BSA, MSA, Heparin, PVA (0-600 μM), and glycerol (0-50% percent) in PBS buffer (10 mM, pH 7.4) at room temperature. λ_{ex} = 580 nm.

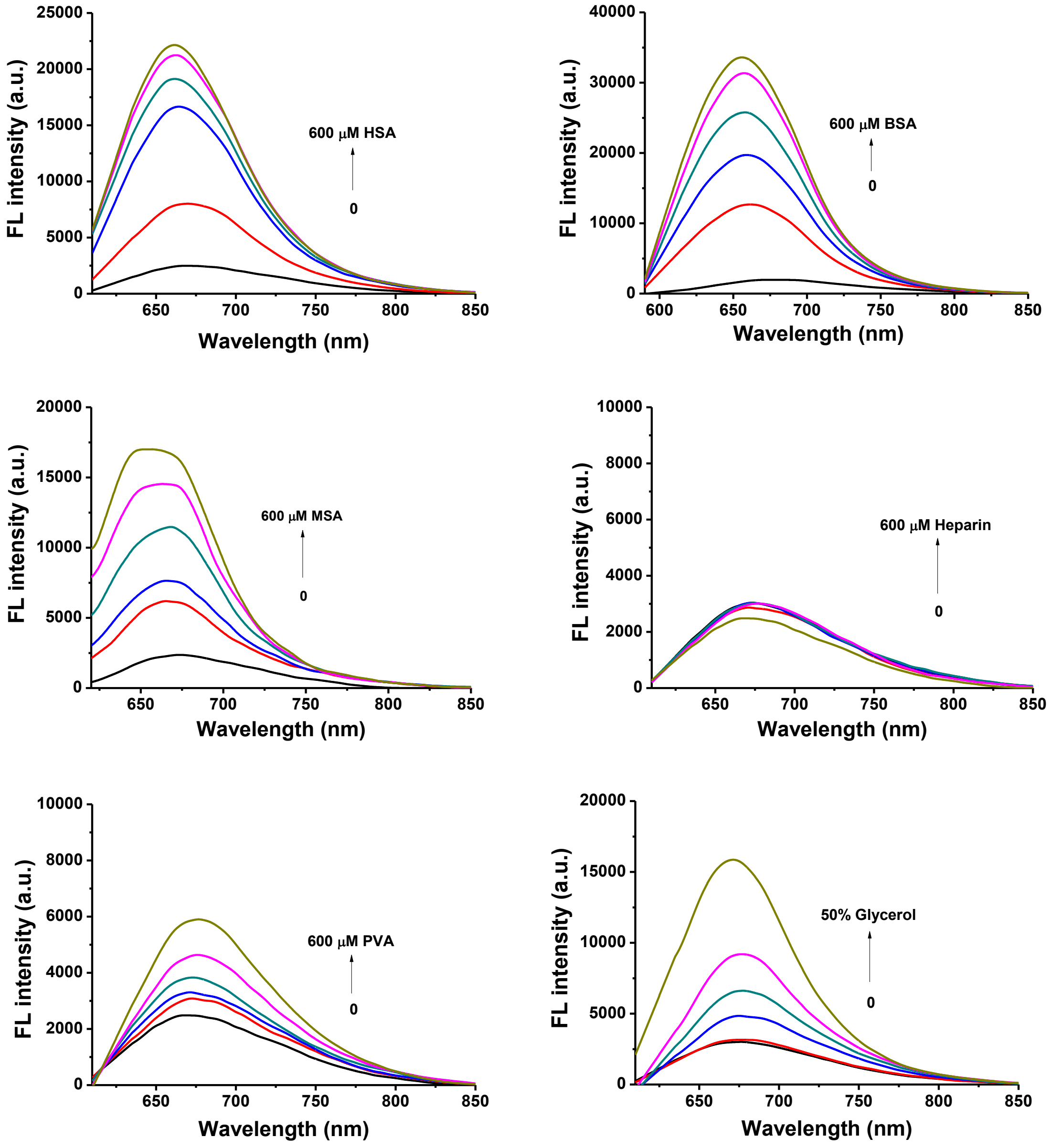

Supplementary Figure 2. Fluorescence examination of NLB. Fluorescence intensity changes of NLB (10 μM) upon addition of HSA, BSA, MSA, Heparin, PVA (0-600 μM), and glycerol (0-50% percent) in PBS buffer (10 mM, pH 7.4) at room temperature. λ_{ex} = 600 nm.

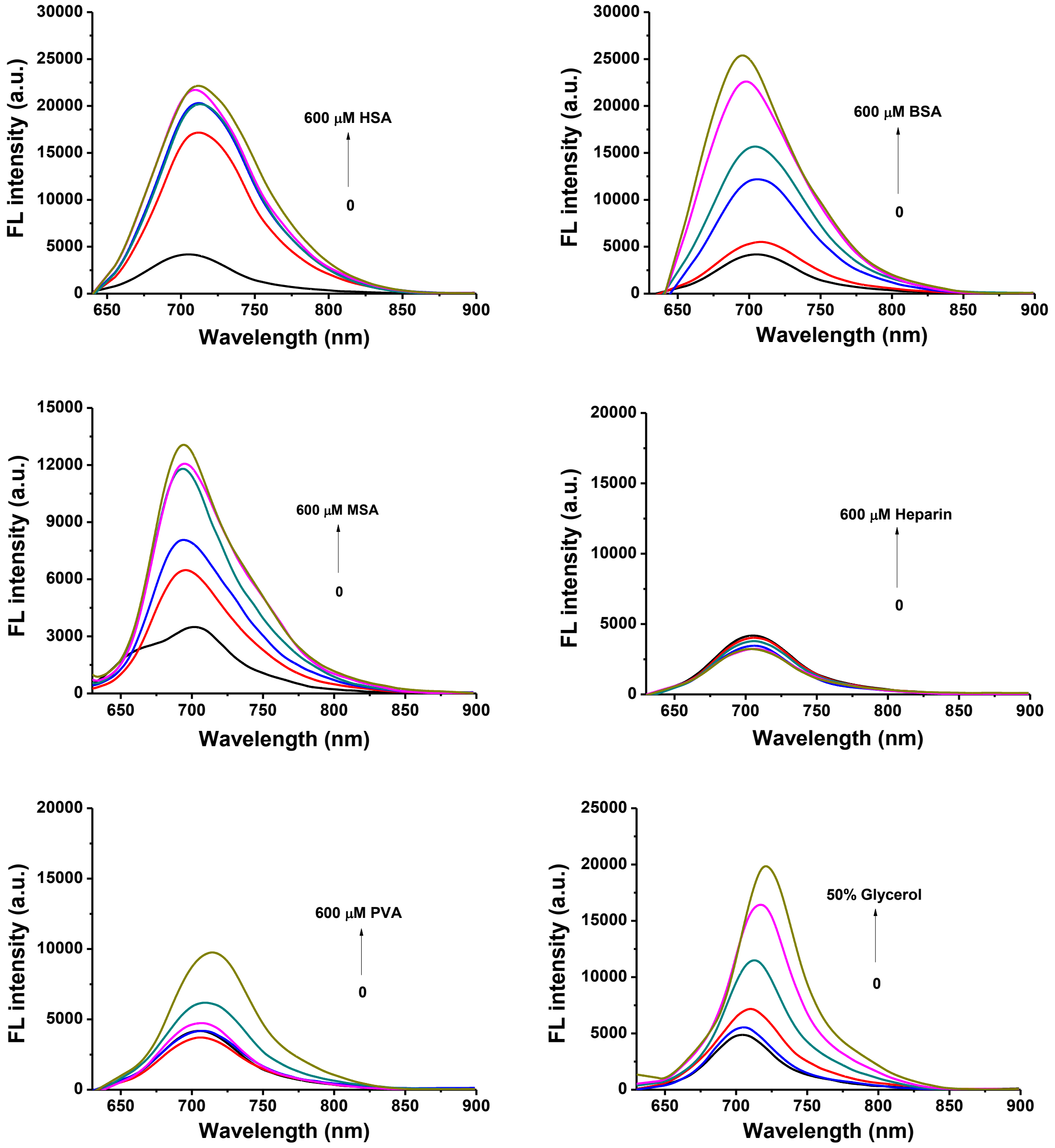

Supplementary Figure 3. Fluorescence examination of HCy7. Fluorescence intensity changes of HCy7 (10 μM) upon addition of HSA, BSA, MSA, Heparin, PVA (0-600 μM), and glycerol (0-50% percent) in PBS buffer (10 mM, pH 7.4) at room temperature. λ_{ex} = 620 nm.

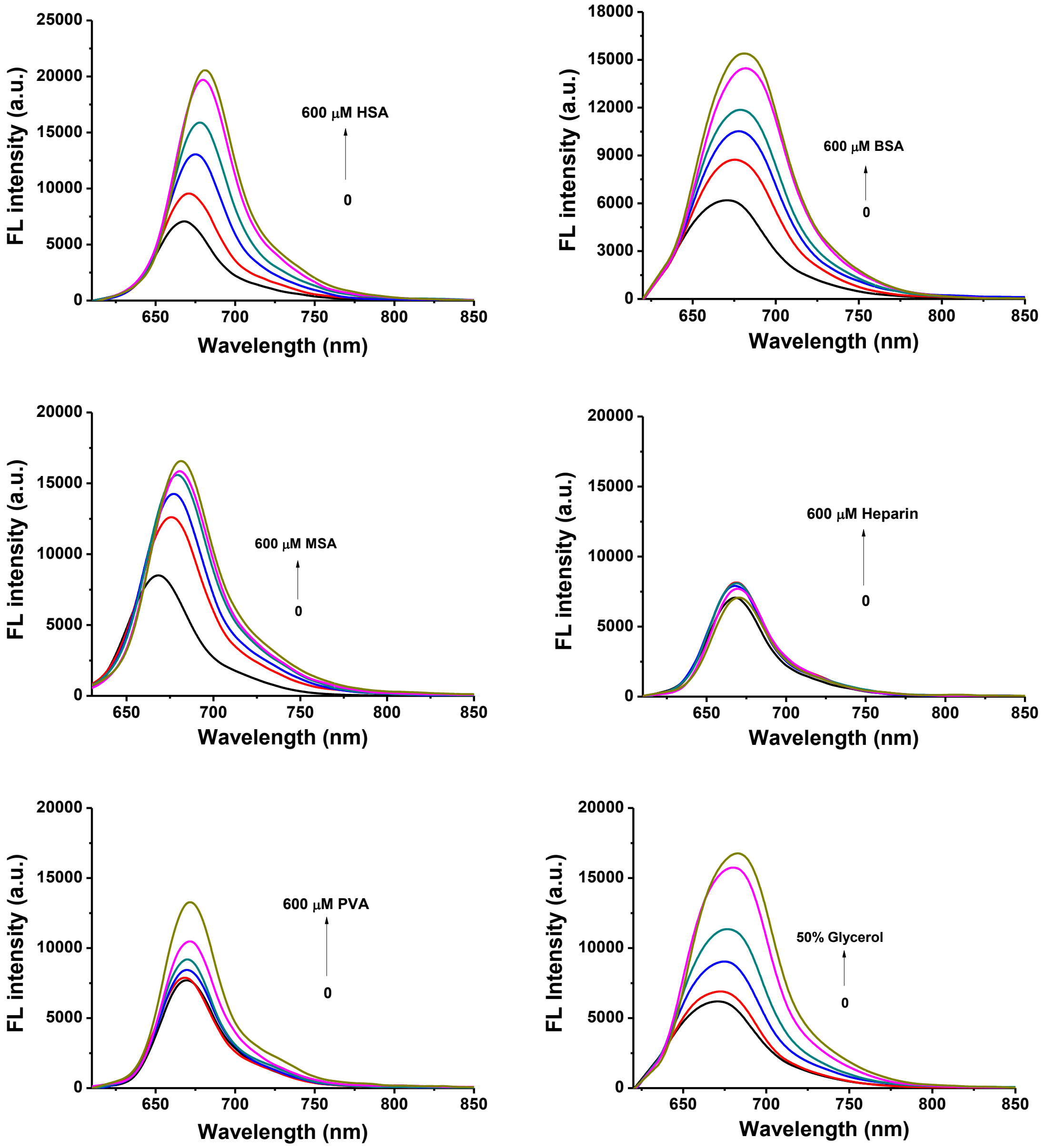

Supplementary Figure 4. Fluorescence examination of Cy5. Fluorescence intensity changes of Cy5 (10 μM) upon addition of HSA, BSA, MSA, Heparin, PVA (0-600 μM), and glycerol (0-50% percent) in PBS buffer (10 mM, pH 7.4) at room temperature. λ_{ex} = 600 nm.

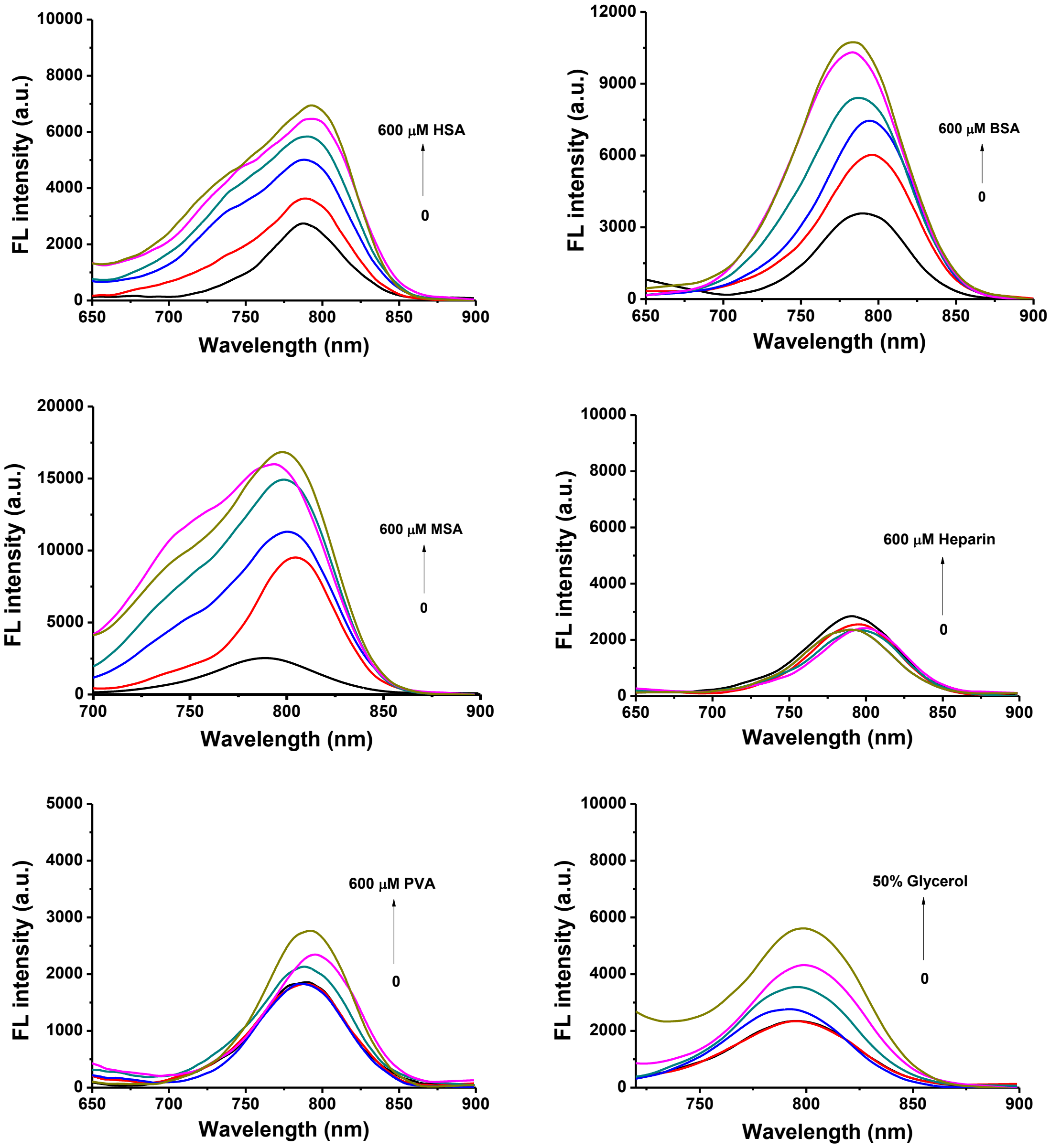

Supplementary Figure 5. Fluorescence examination of Cy7. Fluorescence intensity changes of Cy7 (10 μM) upon addition of HSA, BSA, MSA, Heparin, PVA (0-600 μM), and glycerol (0-50% percent) in PBS buffer (10 mM, pH 7.4) at room temperature. λ_{ex} = 630 nm.

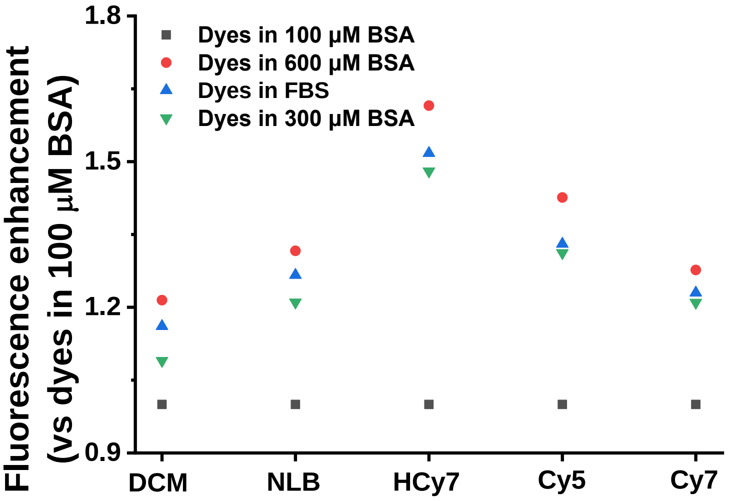

Supplementary Figure 6. Fluorescence enhancement of DCM, NLB, Cy5, HCy7, and Cy7 (10 μM for each) in different concentration of BSA (300, 600 μM) and FBS. The data was determined by the ratio between the fluorescence intensity of dyes in 300, 600 μM BSA, FBS and the intensity of dyes in 100 μM BSA. Source data are provided as a Source Data file.

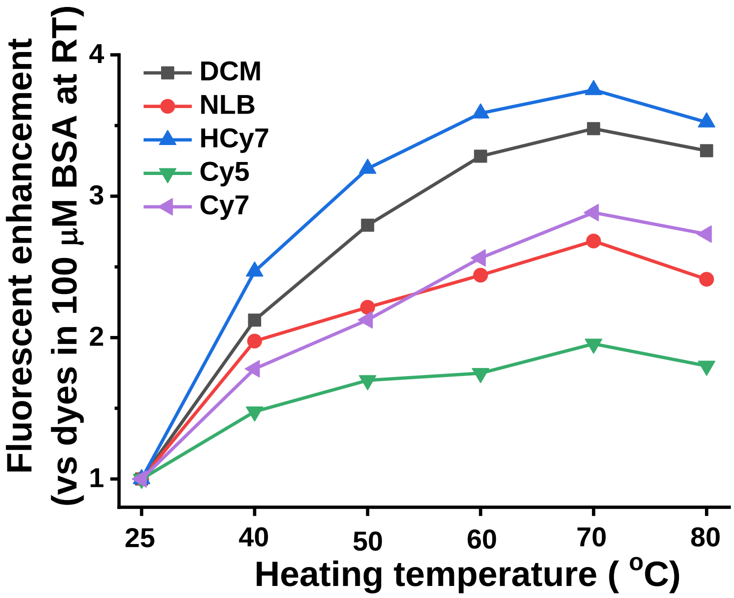

Supplementary Figure 7. Fluorescence enhancement of DCM, NLB, Cy5, HCy7, and Cy7 (10 μM for each) premixed with 100 μM BSA at different temperatures. Each spectrum was recorded after heating for 10 min. Source data are provided as a Source Data file.

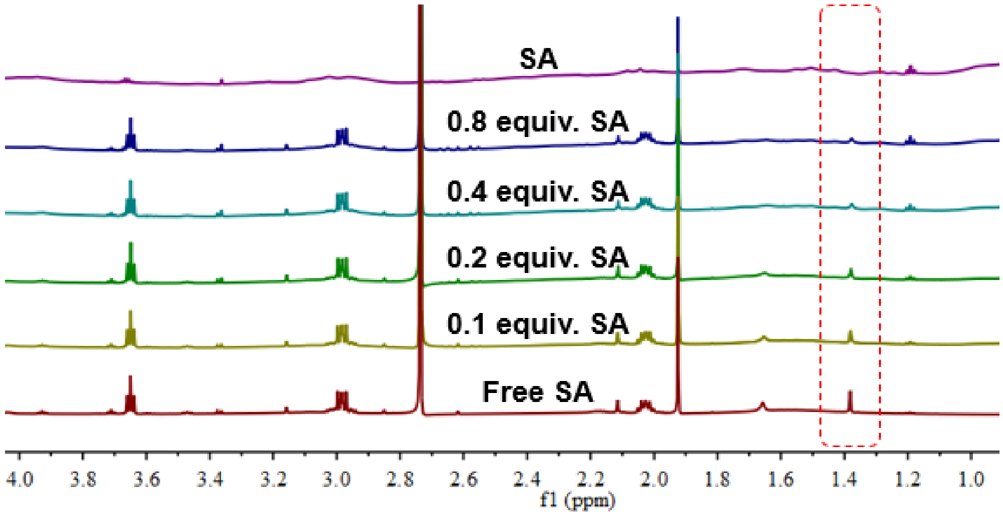

Supplementary Figure 8. ^{1}H NMR titration of different equivalent albumin (0.1 to 0.8 equiv.) to Cy5S in D_2O (1-4 ppm). Red frames highlight the proton signals on the A group.

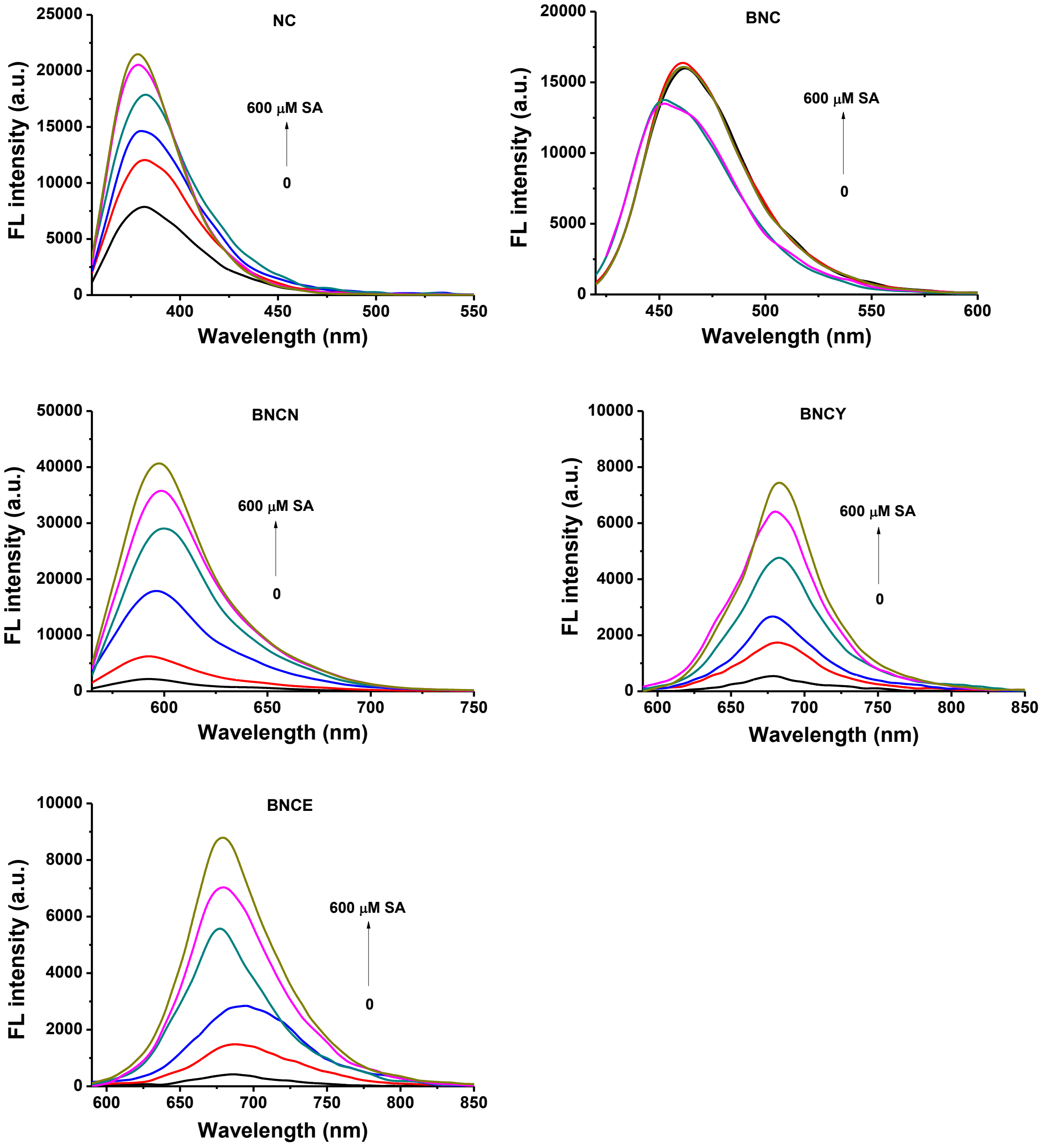

Supplementary Figure 9. Fluorescence intensity changes of NC, BNC, BNCN, BNCY, and BNCE (10 μM) upon addition of SA (0-600 μM) in PBS buffer (10 mM, pH 7.4) at room temperature.

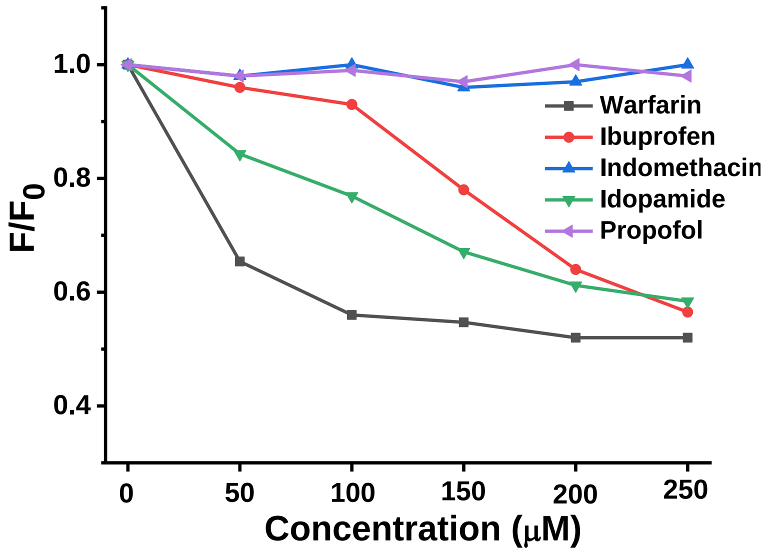

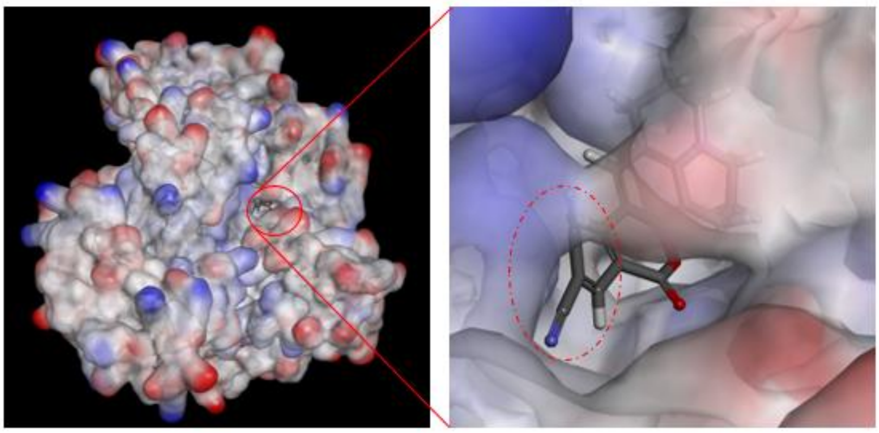

Supplementary Figure 10. Competitive assay to BNCN (10 μM) with SA (10 μM) in PBS buffer solution (pH 7.4, 10 mM) at room temperature. And the conformation of BNCN in site IA.

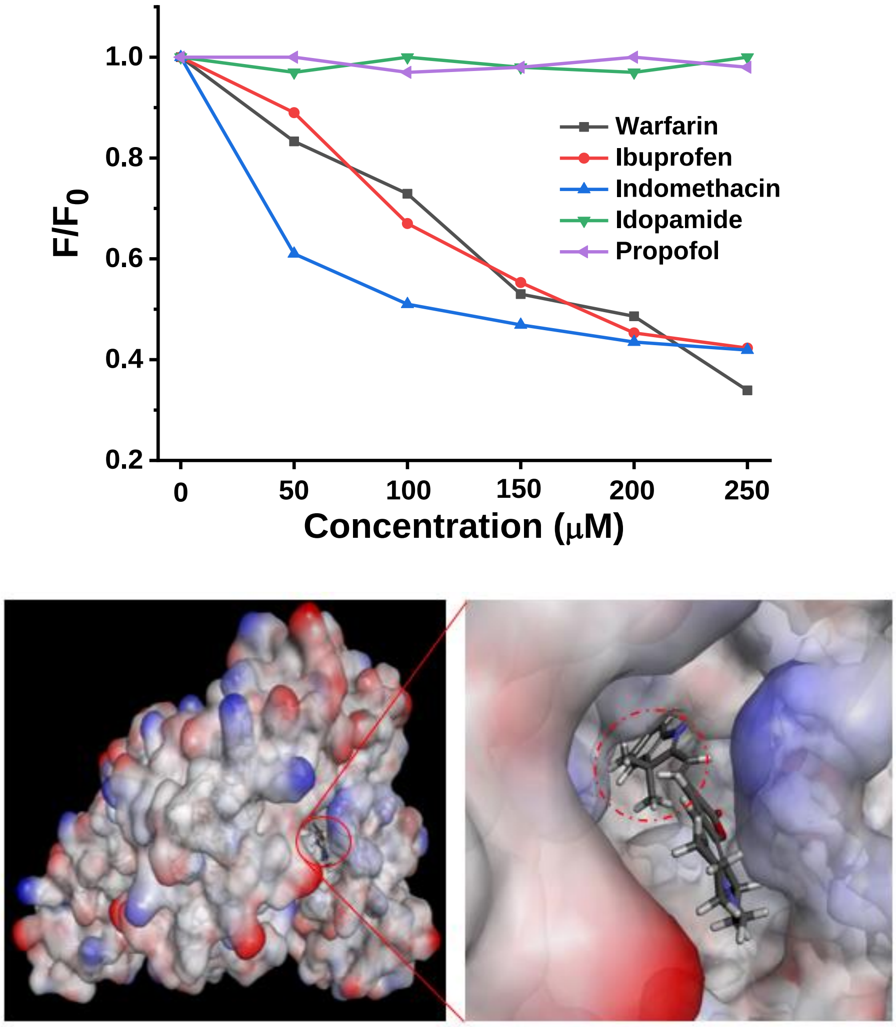

Supplementary Figure 11. Competitive assay to BNCY (10 μM) with SA (10 μM) in PBS buffer solution (pH 7.4, 10 mM) at room temperature. And the conformation of BNCY in site IA.

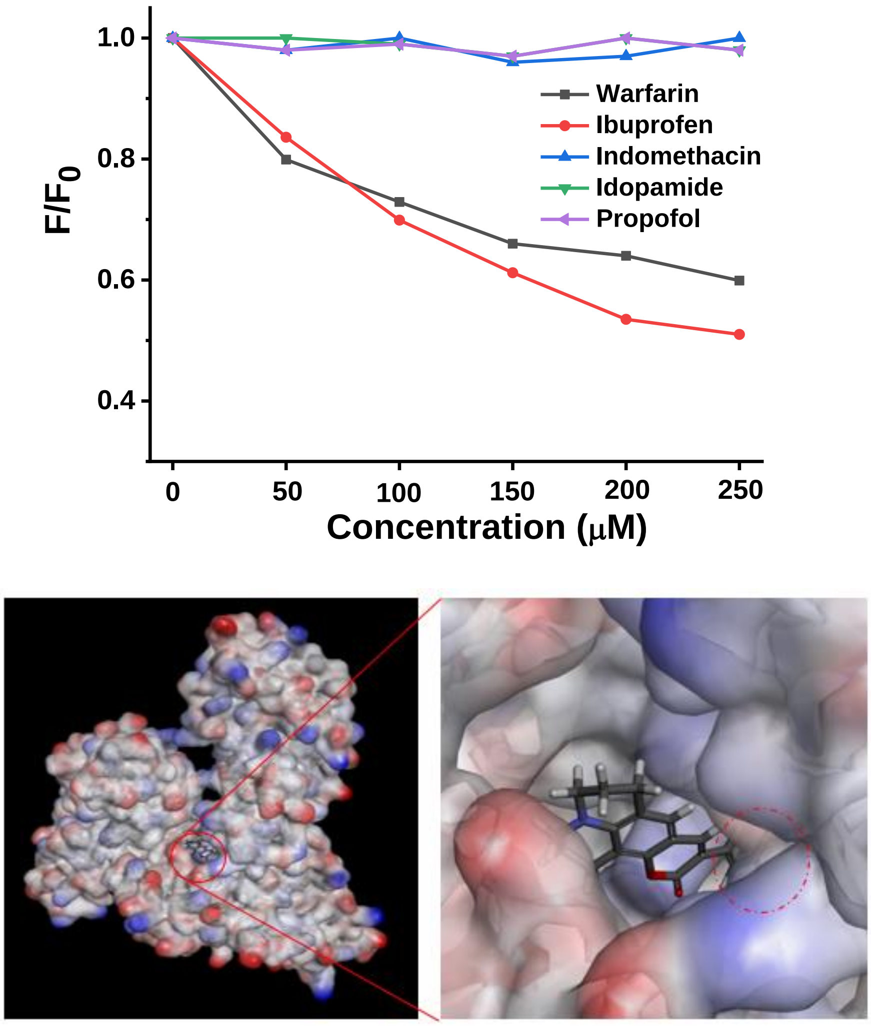

Supplementary Figure 12. Competitive assay to BNCE (10 μM) with SA (10 μM) in PBS buffer solution (pH 7.4, 10 mM) at room temperature. And the conformation of BNCE in site IA.

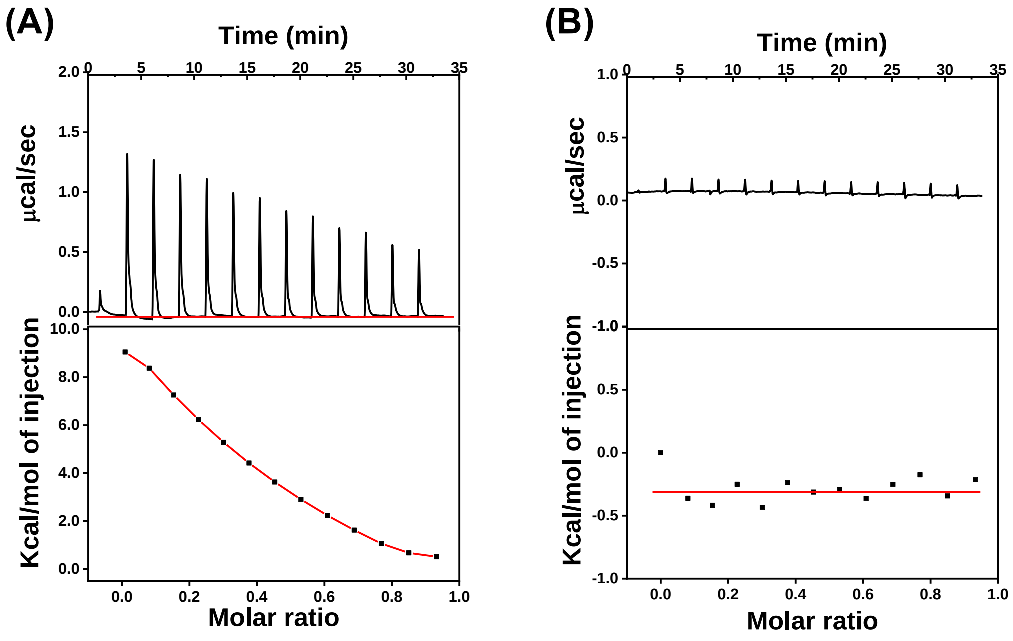

Supplementary Figure 13. Quantitative measurements of NLB and BNLB binding to albumin. (A) Quantification of NLB binding to albumin by ITC. Albumin (500 μM in the syringe) was injected into a 100 μM solution of NLB. The data were fitted with a simple one-site-binding model, yielding a Kd of 161 μM. (B) Quantification of BNLB binding to albumin by ITC. Albumin (500 μM in the syringe) was injected into a 100 μM solution of BNLB. No binding was observed. Condition: PBS (10 mM, pH 7.4, containing 5% DMSO, v/v).

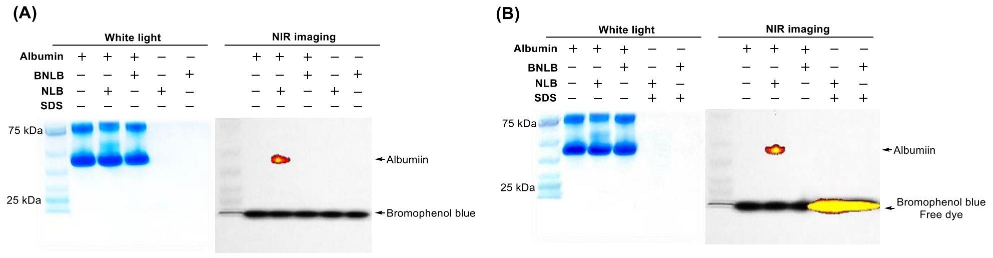

Supplementary Figure 14. Electrophoresis analysis of NLB and BNLB binding to albumin on 8% native PAGE. The gels were imaged under fluorescence (red band) and then stained with Coomassie brilliant blue to visualize proteins (blue band). Comparison of the addition of SDS (A) or not (B) in the electrophoresis of free dyes. Data are representative for three independent experiments. Source data are provided as a Source Data file.

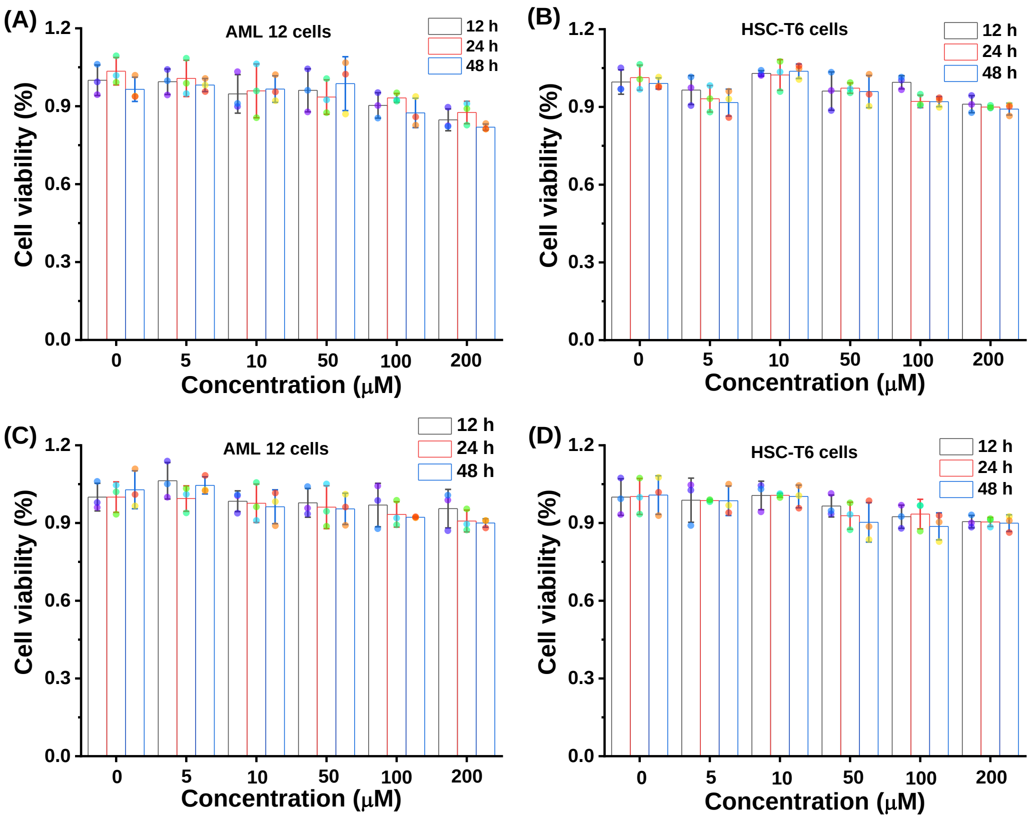

Supplementary Figure 15. Concentration dependent cytotoxicity of NLBN (A and B) and BNLBN (C and D) to AML 12 cells and HSC-T6 cells at 12, 24, and 48 h determined by CCK-8 assay. Data are represented as mean values ± SD (n=3 independent cell samples). Source data are provided as a Source Data file.

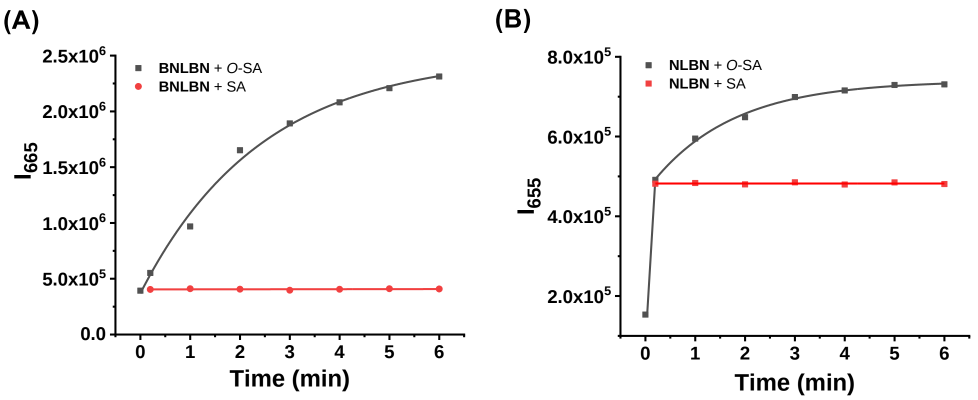

Supplementary Figure 16. Time-course of BNLBN (A) and NLBN (B) with the addition of *O*-SA and SA. Reaction conditions: 10 μM probe was mixed with 5 μM *O*-SA/SA in a PBS (10 mM, pH 7.4) solution.

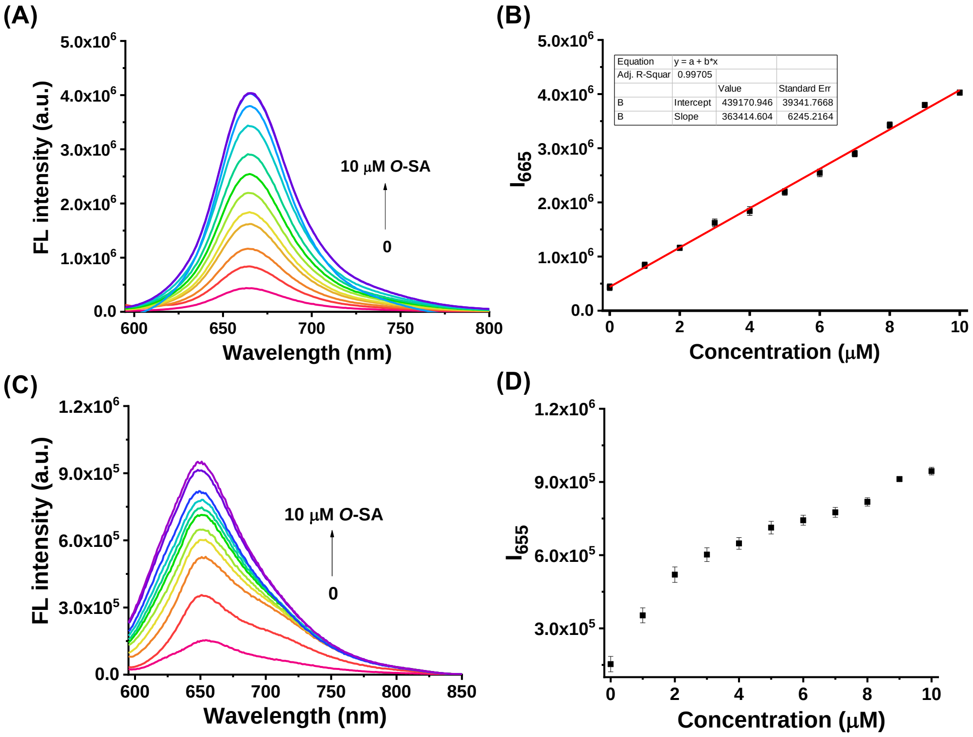

Supplementary Figure 17. Fluorescent titration of BNLBN and NLBN. (A) Fluorescent spectrum of BNLBN (10 μM) with the addition of *O*-SA (0-10 μM) in PBS (10 mM, pH 7.4) and (B) the linear relationship between them; (C) Fluorescent spectrum of NLBN (10 μM) with the addition of *O*-SA (0-10 μM) in PBS (10 mM, pH 7.4) and (D) the linear relationship between them. Each spectrum was recorded after 5 min. Data are represented as mean values ± SD (n=3 independent experiments).

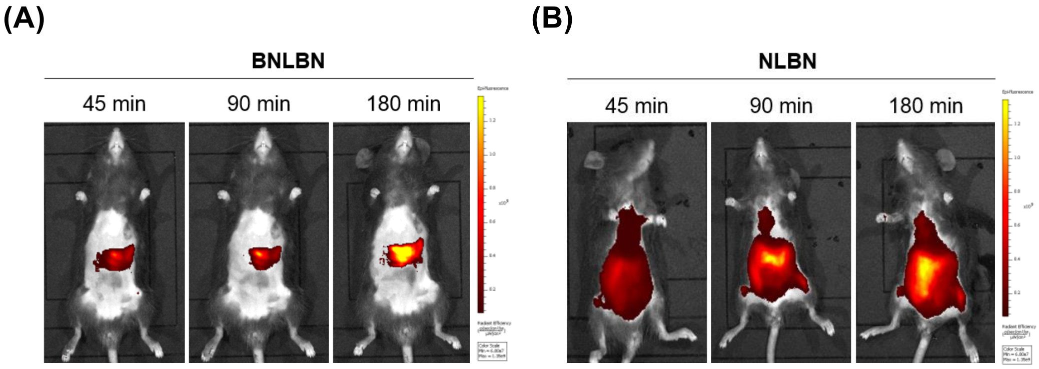

Supplementary Figure 18. Time-dependent imaging *in vivo*. BNLBN (A) and NLBN (B; 200 μM in a 10 mM PBS solution, 100 μL for each) were injected *via* the tail vein. And images were captured at 45, 90, and 180 min.

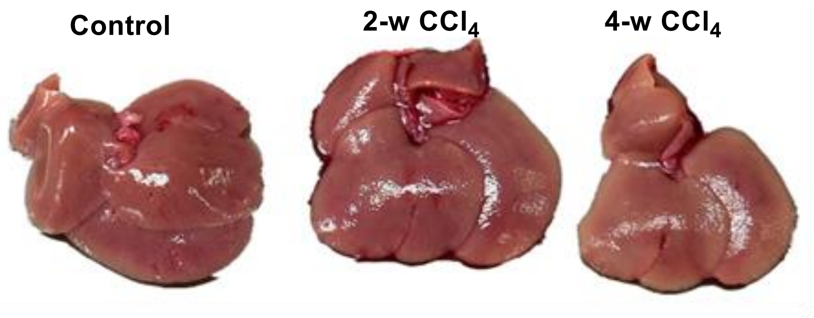

Supplementary Figure 19 Morphological comparison of livers between the control and CCl_4 administered model groups.

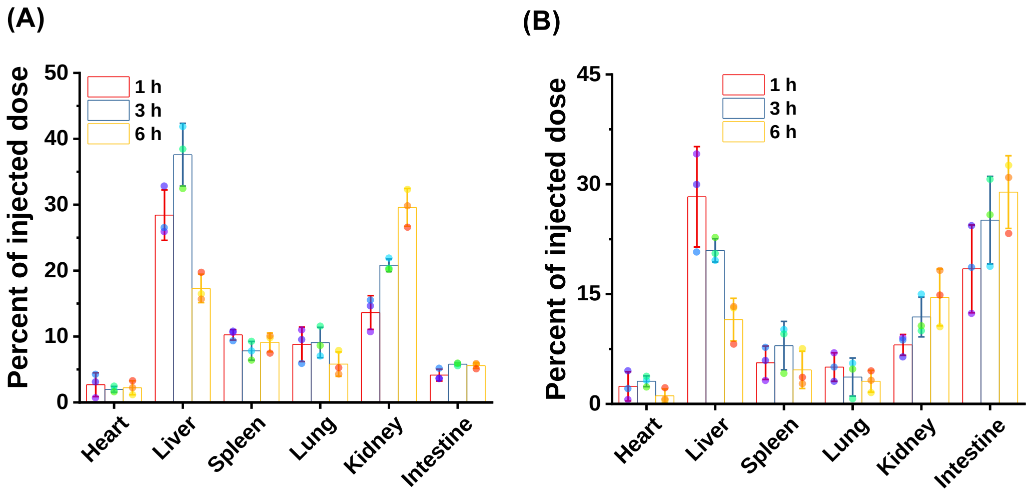

Supplementary Figure 20. Quantitative analysis of BNLBN and NLBN in five major organs (heart, liver, spleen, lung, and kidney) at 1, 3, and 6 h post-injection. Data are represented as mean values ± SD (n=3 independent tissue samples per group).

(A)

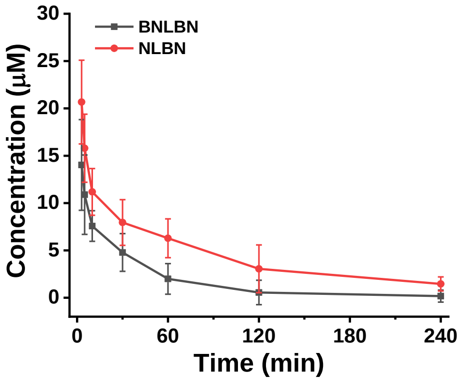

(B)

Pharmacokinetics	BNLBN	NLBN
$t_{1/2\alpha}$ (min)	1.64	1.55
$t_{1/2\beta}$(min)	28.75	78.08
K (min^{-1})	0.061	0.037
AUC_{0-t} (mg·min/mL)	197.49	451.43
$AUC_{0-\infty}$ (mg·min/mL)	217.69	539.01

Supplementary Figure 21. Pharmacokinetics of BNLBN and NLBN in mice. (A) Time–concentration curves from 3 to 240 min. Data are represented as mean values ± SD (n=3 mice for each group); (B) Pharmacokinetic parameters in the blood. AUC, area under drug concentration-time curve. Source data are provided as a Source Data file.

(A)

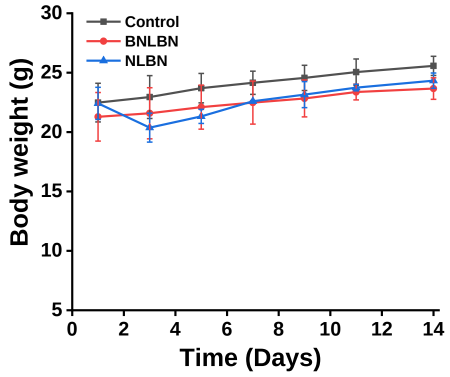

(C)

	BUN (mM/L)	LDH (U/L)	AST (U/L)	ALT (U/L)
Control	9.0 ± 0.7	534.0 ± 4.7	28.2 ± 3.6	14.5 ± 1.7
**BNLBN**	9.6 ± 0.6	562.9 ± 3.2	25.5 ± 3.5	14.7 ± 3.3
**NLBN**	10.2 ± 1.4	545.1 ± 2.8	21.2 ± 3.8	17.1 ± 2.3

(B)

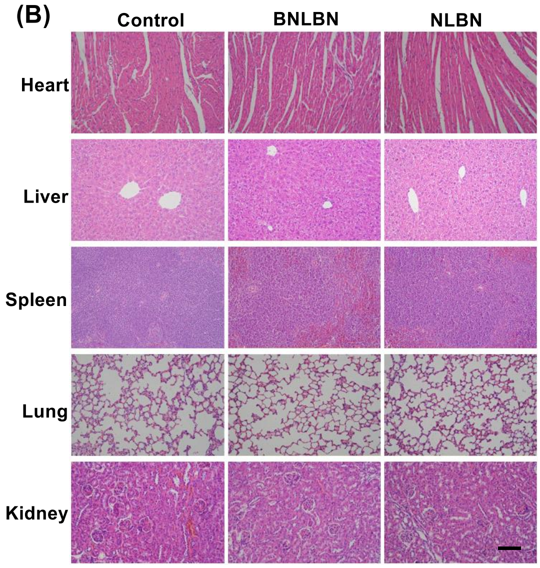

Supplementary Figure 22. *In vivo* toxicity study of BNLBN and NLBN. (A) Mice were weighted every three days after the injection of probes (200 μM, 100 μL for each). Data are represented as mean values ± SD (n=3 mice for each group); (B) Clinical chemistry indexes analysis of BUN, LDH, AST, and ALT. Data are representative for three independent animals; (C) H&E stained tissue slices (heart, liver, spleen, lung, and kidney). Scale bar in the image is 100 μm.

Supplementary Table 1. Binding energy for NC, BNCN, BNCY, and BNCE with albumin pockets.

		NC	BNCN	BNCY	BNCE
Site I	-CDOCKER ENERGY	30.877	-17.0063	20.7743	---
	-CDOCKER INTERACTION ENERGY	49.9449	42.6668	36.228	---
Site IIA	-CDOCKER ENERGY	29.2858	21.4682	-1.15496	-12.8607
	-CDOCKER INTERACTION ENERGY	50.5007	33.5647	39.1084	39.3232
Site IIB	-CDOCKER ENERGY	30.6378	---	-22.0613	19.3894
	-CDOCKER INTERACTION ENERGY	52.3211	---	40.6009	41.9951

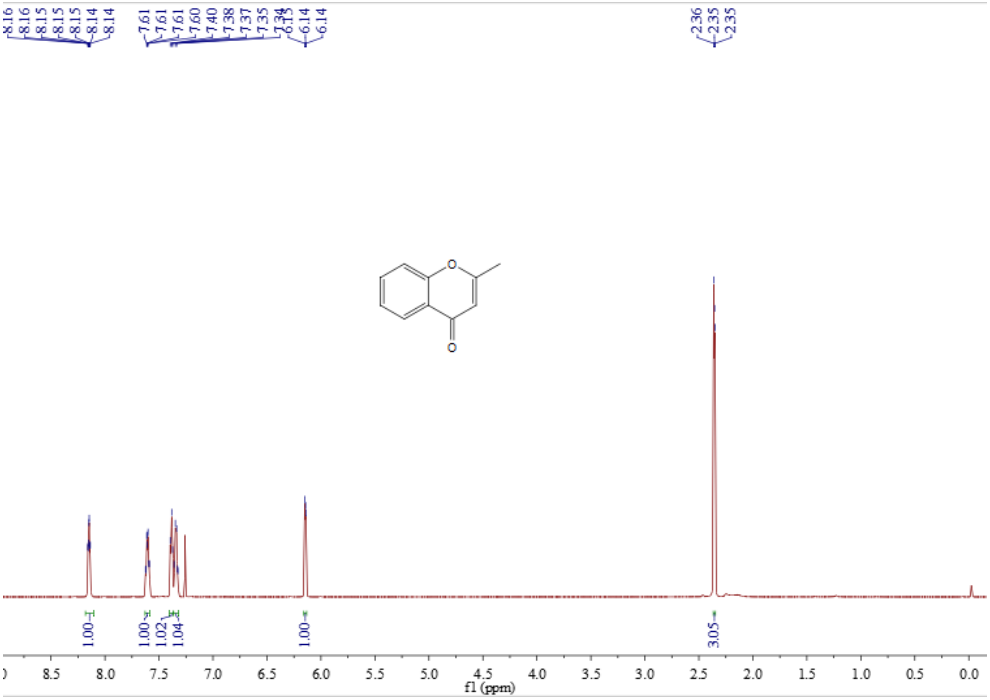

Supplementary Figure 23. ^{1}H NMR of **1-2** in $CDCl_3$.

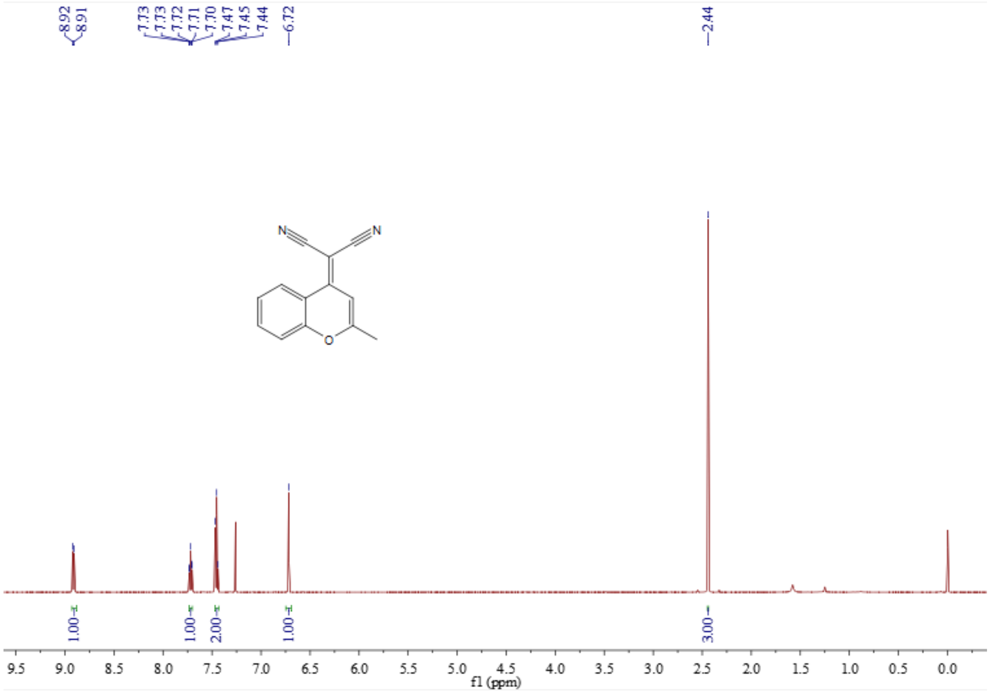

Supplementary Figure 24. ^{1}H NMR of **1-3** in $CDCl_3$.

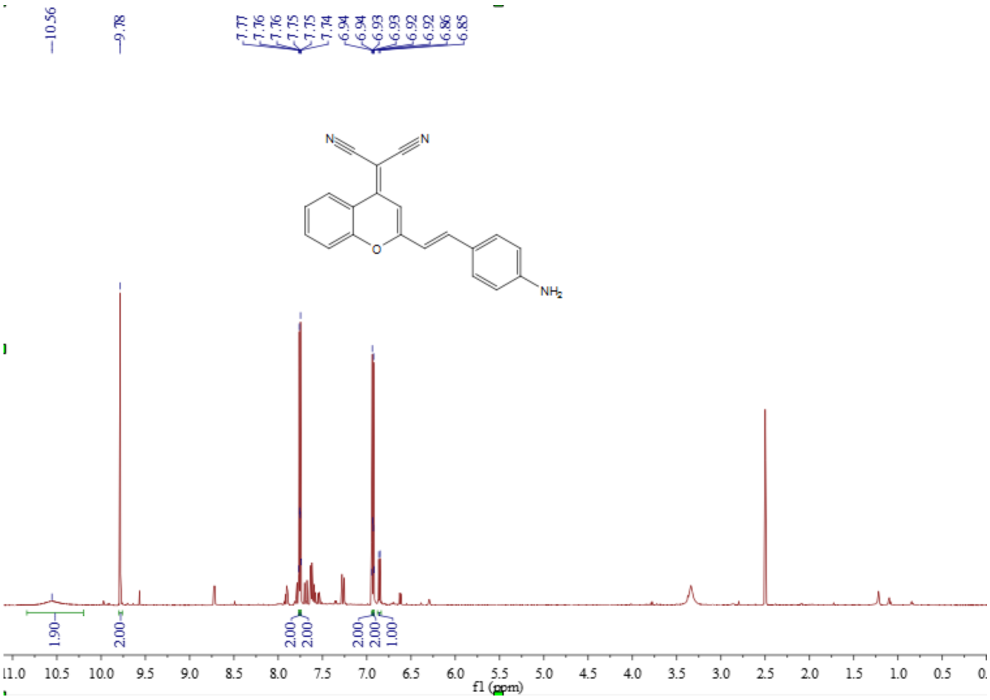

Supplementary Figure 25. ^{1}H NMR of DCM in DMSO-d_6.

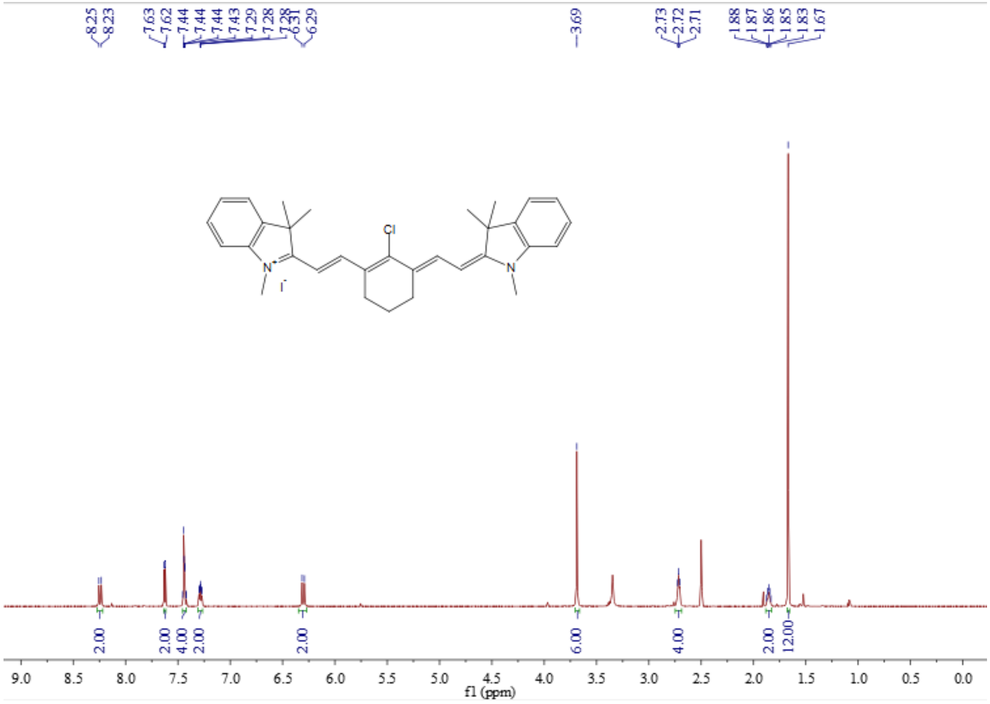

Supplementary Figure 26. ^{1}H NMR of **3-2** in DMSO-d_6.

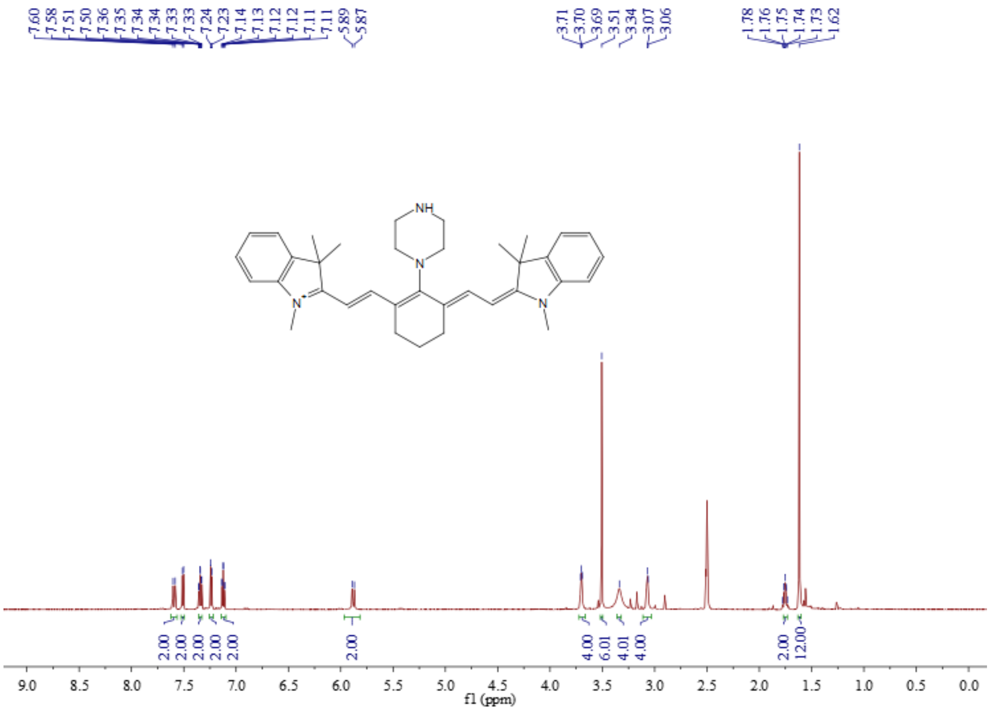

Supplementary Figure 27. ^{1}H NMR of Cy7 in DMSO-d_6.

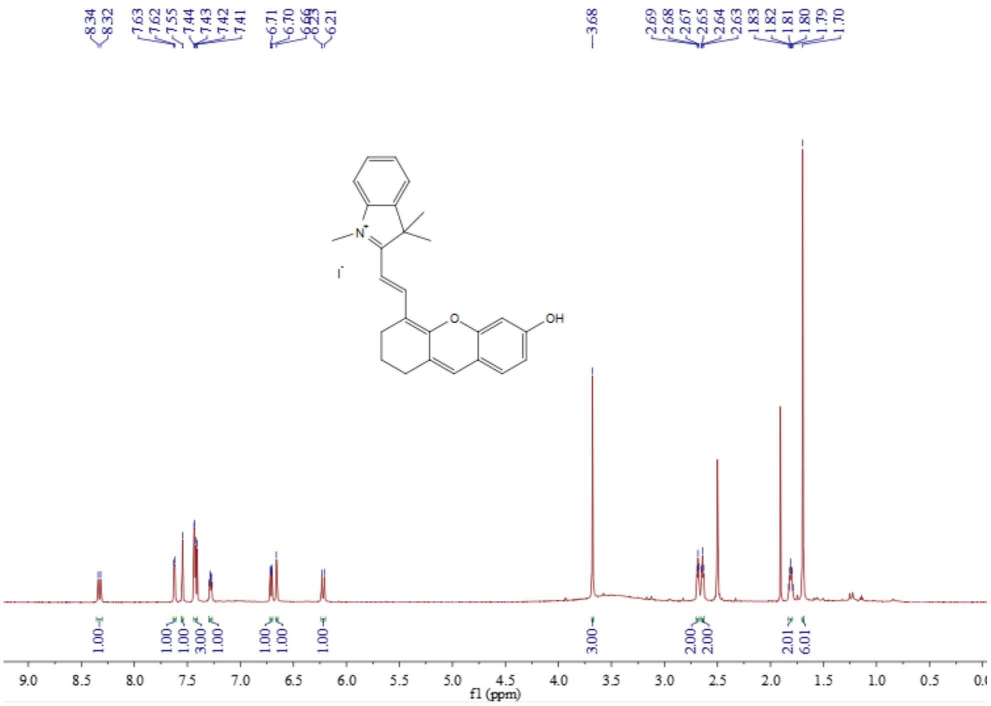

Supplementary Figure 28. ^{1}H NMR of HCy7 in DMSO-d_6.

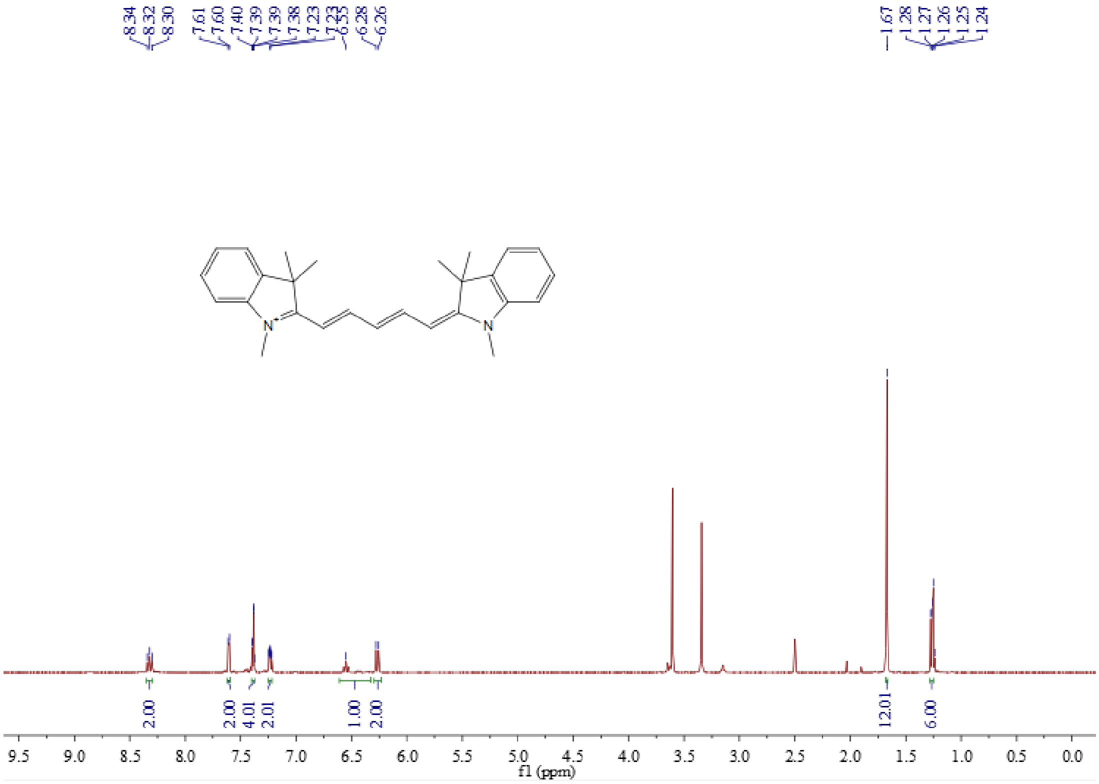

Supplementary Figure 29. ^{1}H NMR of Cy5 in DMSO-d_6.

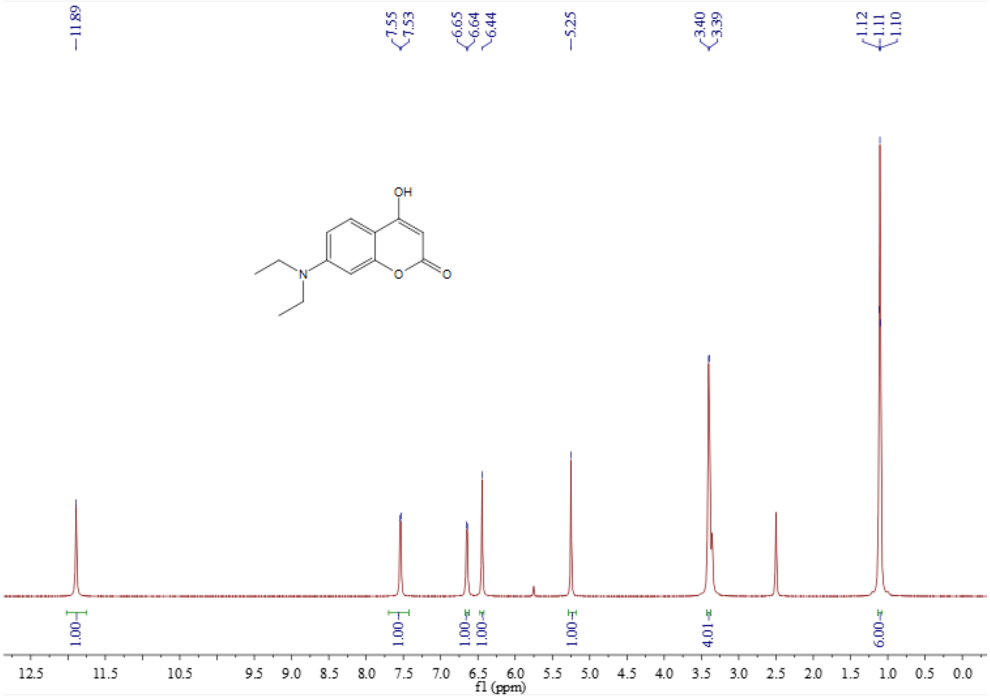

Supplementary Figure 30. ^{1}H NMR of NC in DMSO-d_6.

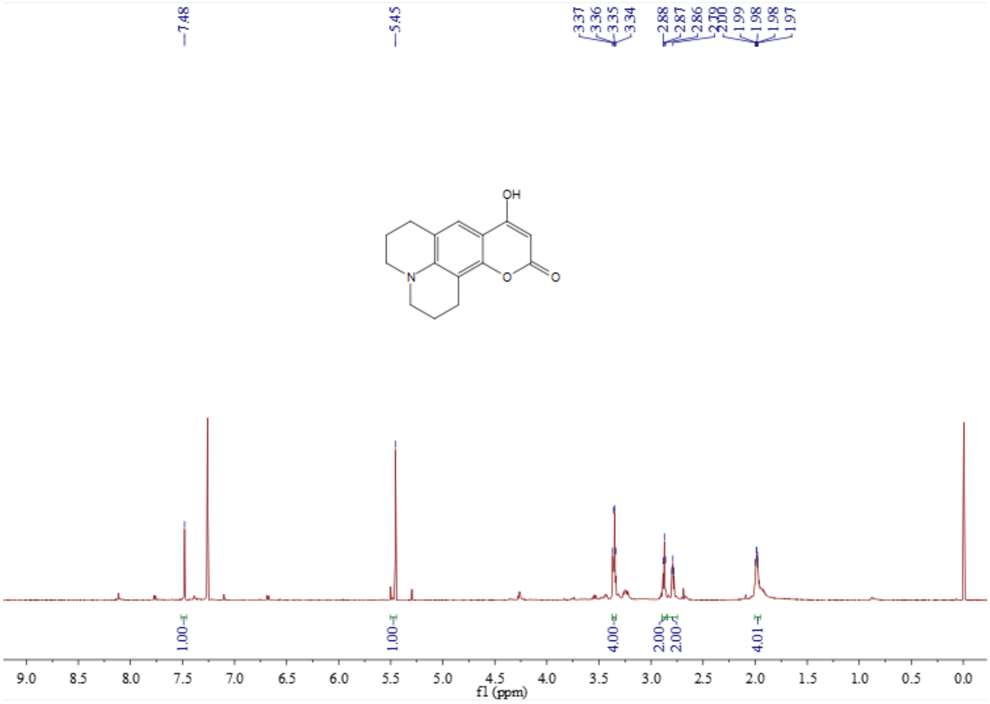

Supplementary Figure 31. ^{1}H NMR of BNC in $CDCl_3$.

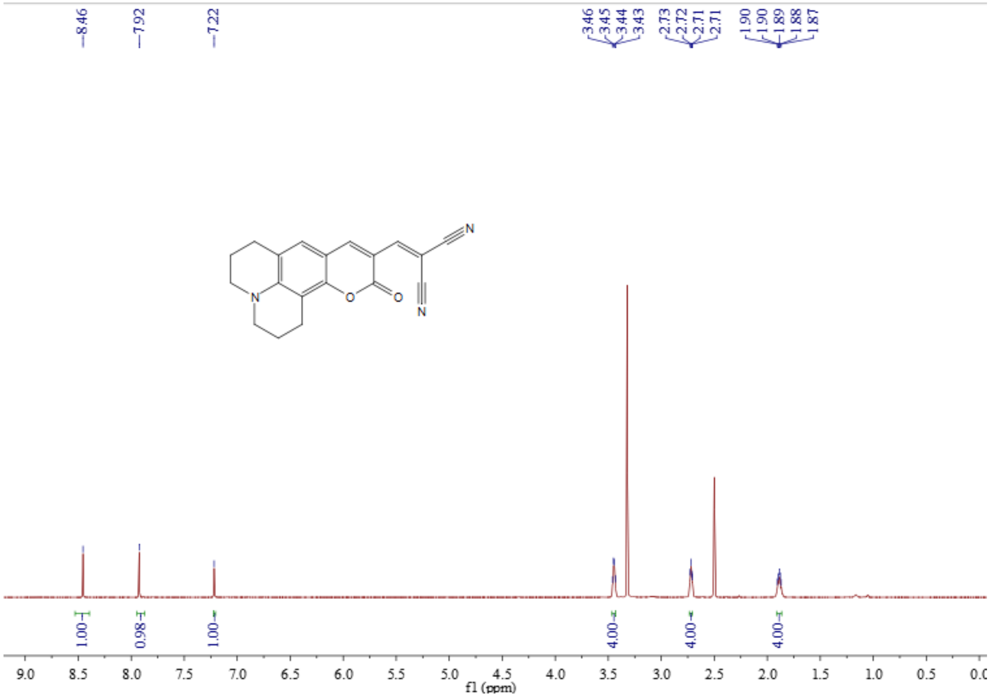

Supplementary Figure 32. ^{1}H NMR of BNCN in DMSO-d_6.

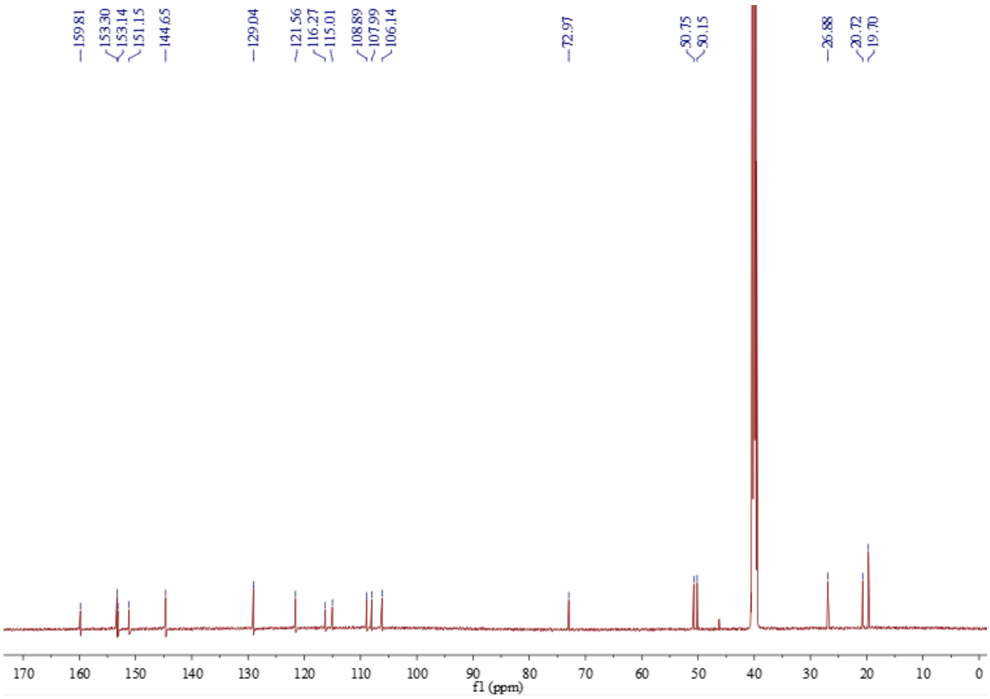

Supplementary Figure 33. ^{13}C NMR of BNCN in DMSO-d_6.

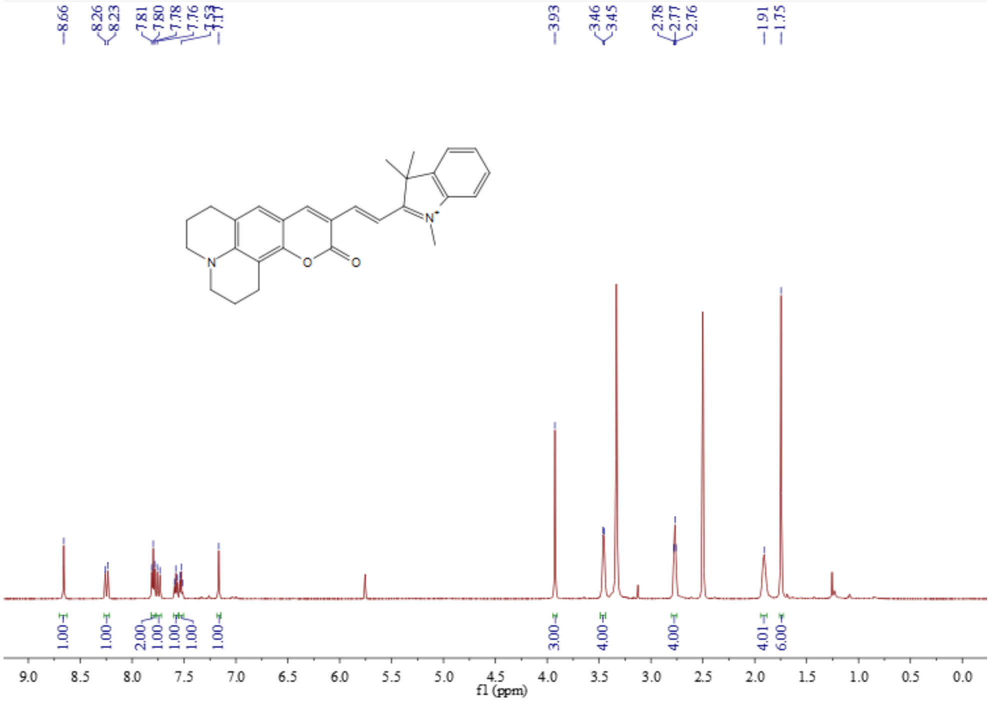

Supplementary Figure 34. ^{1}H NMR of BNCY in DMSO-d_6.

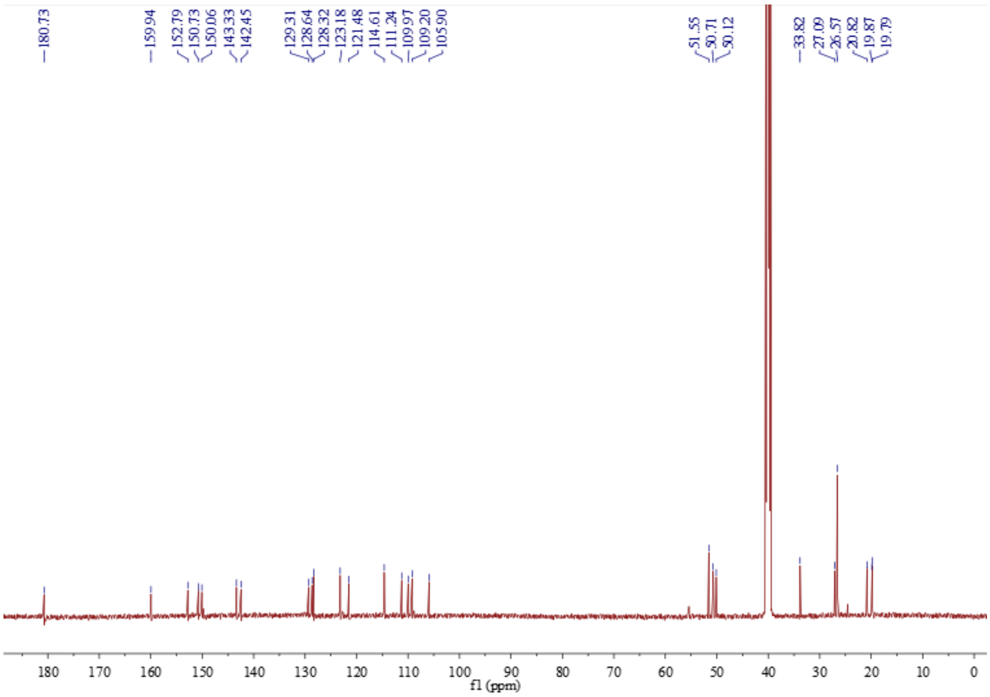

Supplementary Figure 35. ^{13}C NMR of BNCY in DMSO-d_6.

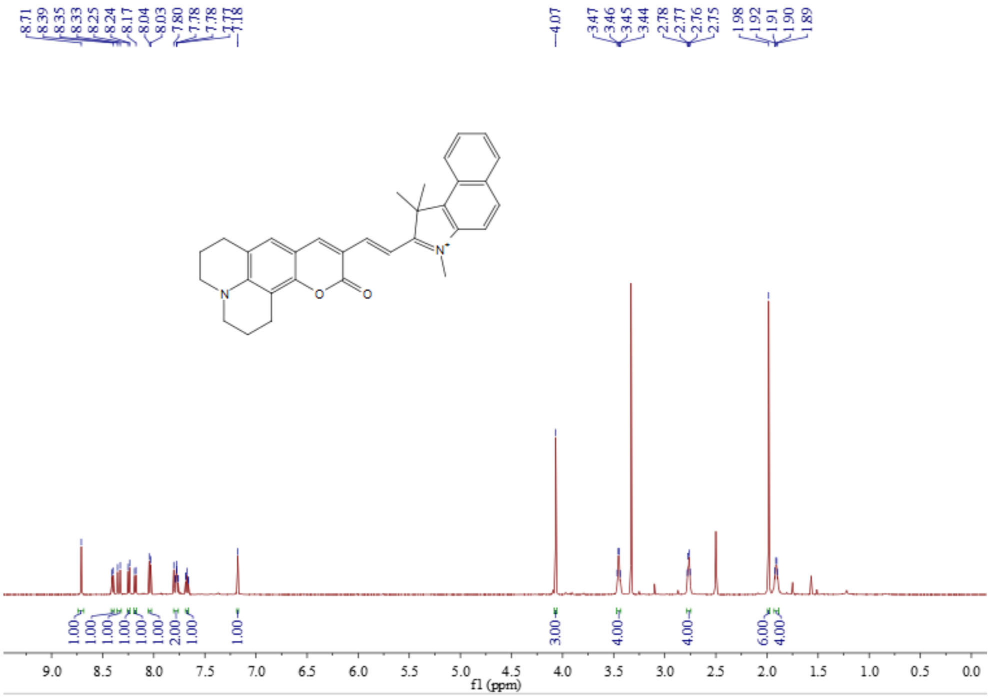

Supplementary Figure 36. ^{1}H NMR of BNCE in DMSO-d_6.

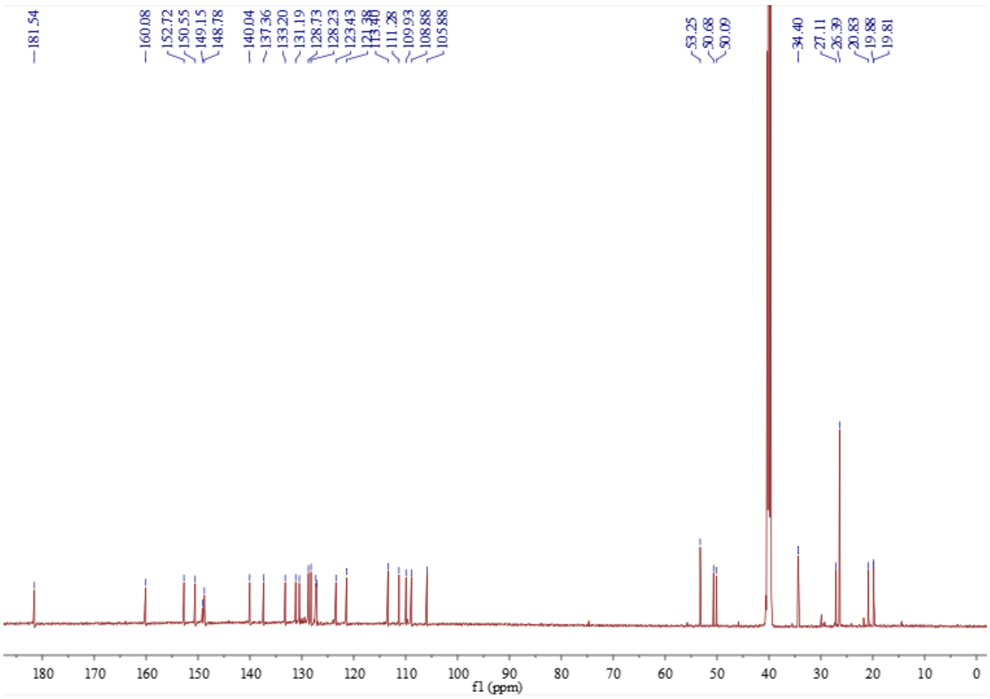

Supplementary Figure 37. ^{13}C NMR of BNCE in DMSO-d_6.

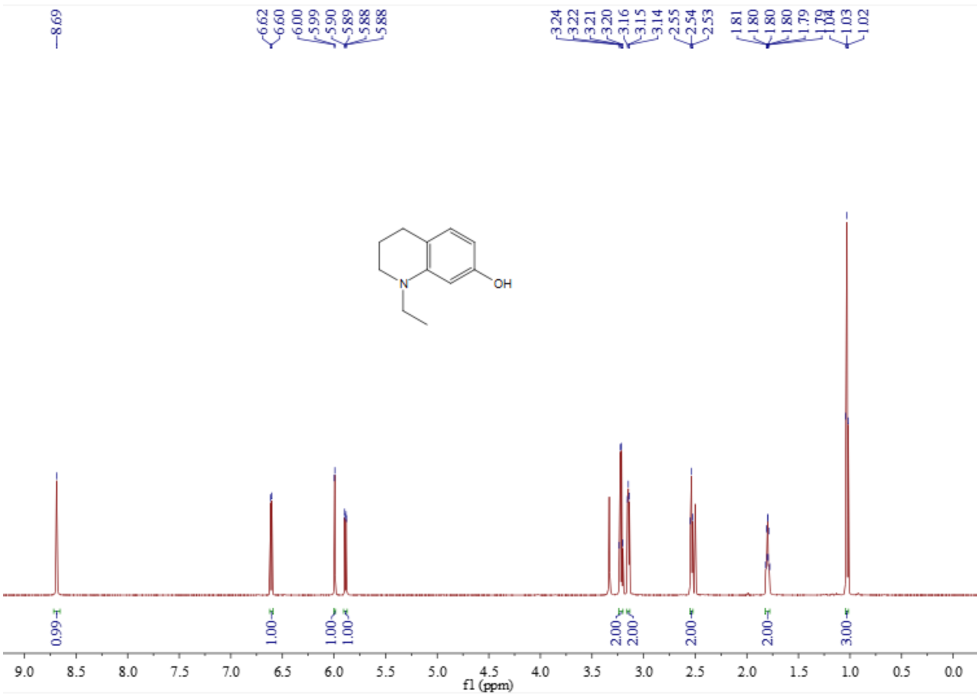

Supplementary Figure 38. ^{1}H NMR of BOH in DMSO-d_6.

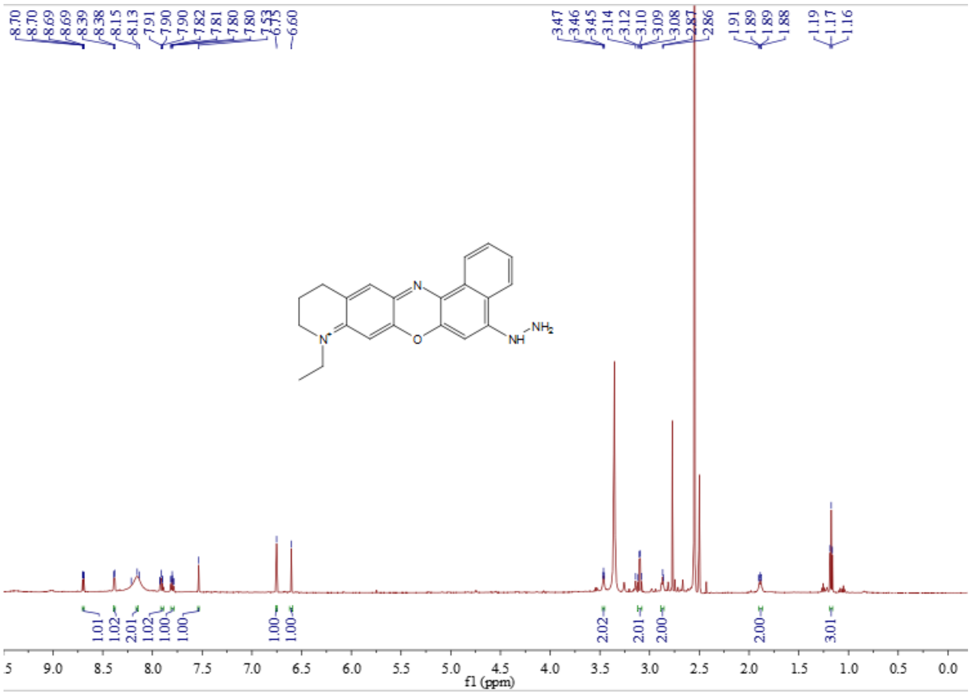

Supplementary Figure 39. ^{1}H NMR of BNLBN in DMSO-d_6.

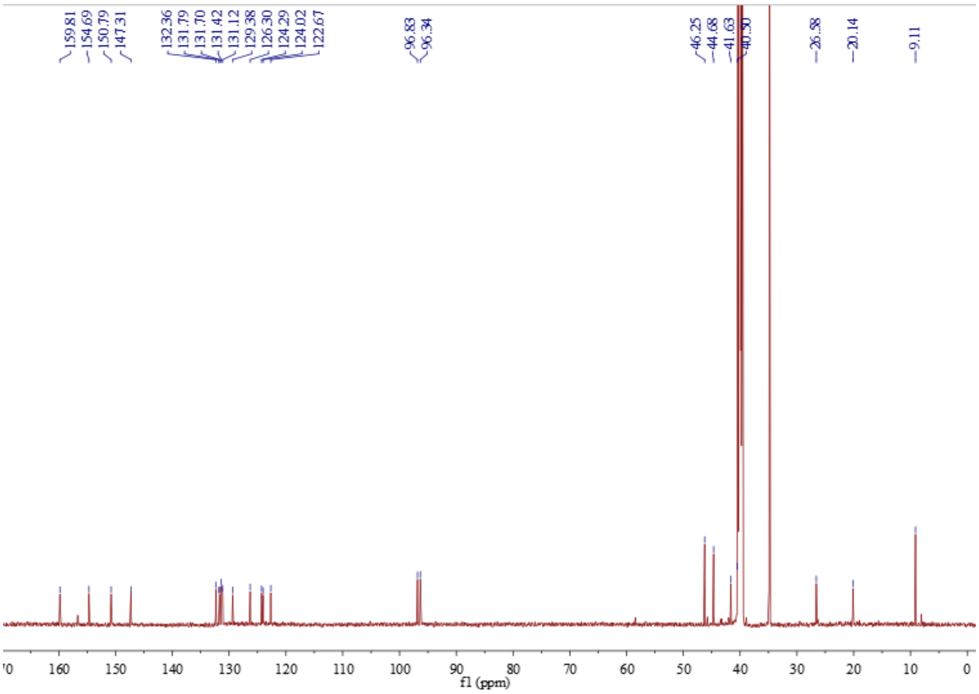

Supplementary Figure 40. ^{13}C NMR of BNLBN in DMSO-d_6.

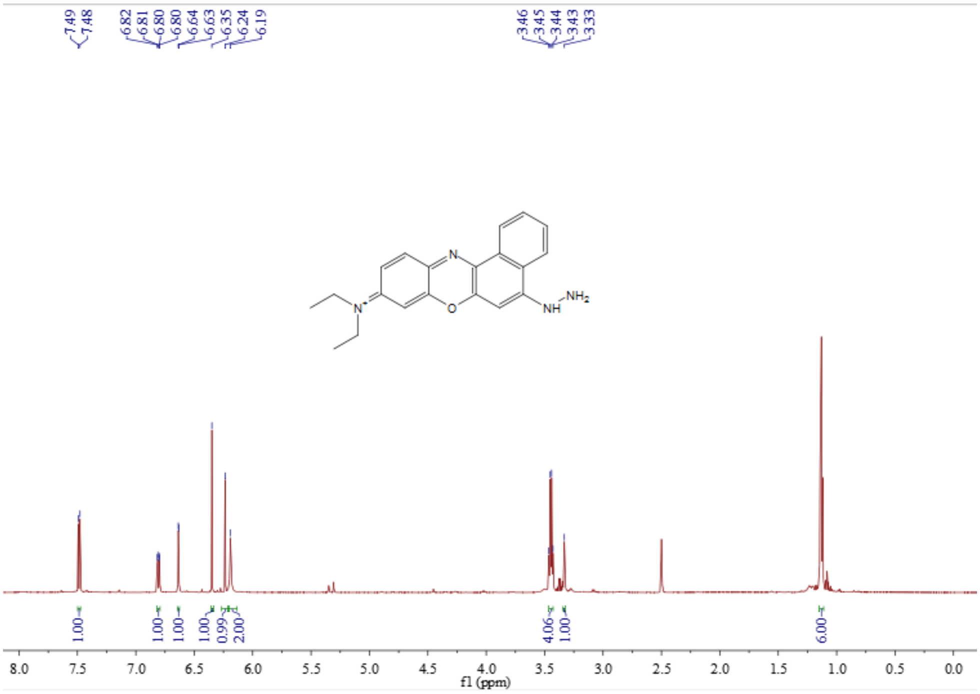

Supplementary Figure 41. ^{1}H NMR of NLBN in DMSO-d_6.

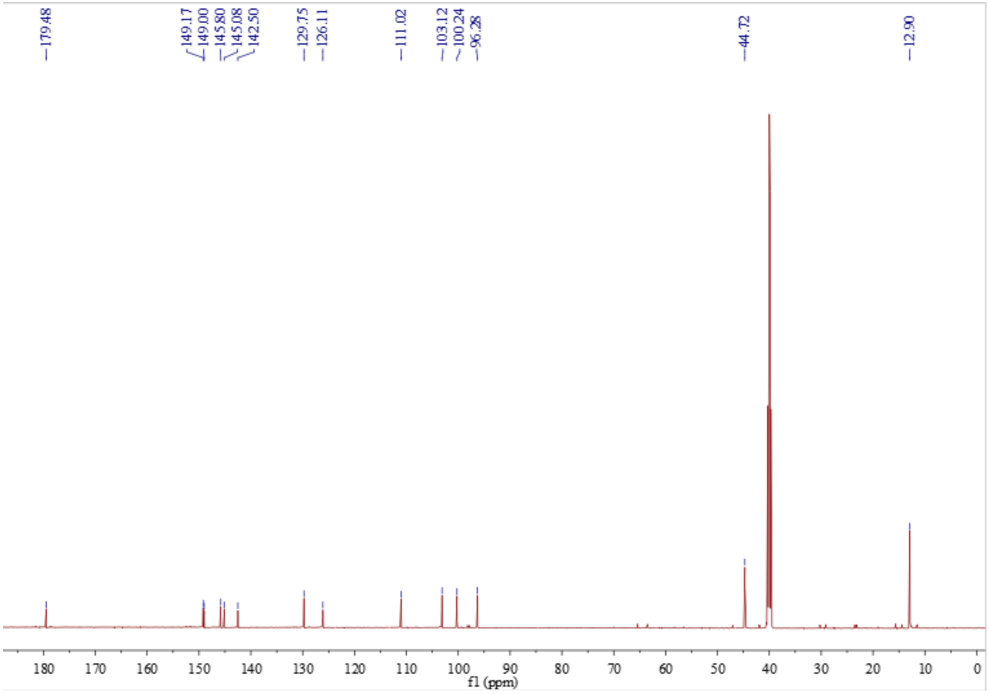

Supplementary Figure 42. ^{13}C NMR of NLBN in DMSO-d_6.

Supplementary References

1. Kong, Y., *et al.* Development of a novel near-infrared fluorescent theranostic combretastain A-4 analogue, YK-5-252, to target triple negative breast cancer. *Bioorgan. Med. Chem.* **25**, 2226-2233 (2017).
2. Wang, K., *et al.* A “turn-on” near-infrared fluorescent probe with high sensitivity for detecting reduced glutathione based on red shift in vitro and in vivo. *Dyes Pigm.* **172**, 107837 (2020).
3. Cao, X., Lin, W. & He, L. A near-infrared fluorescence turn-on sensor for sulfide anions. *Org. Lett.* **13**, 4716-4719 (2011).
4. Zhang, J., *et al.* A novel near-infrared fluorescent probe for sensitive detection of β-galactosidase in living cells. *Anal. Chim. Acta* **968**, 97-104 (2017).
5. Kundu, K., *et al.* Hydrocyanines: a class of fluorescent sensors that can image reactive oxygen species in cell culture, tissue, and in vivo. *Angew. Chem. Int. Edit.* **48**, 299-303 (2009).
6. Davis, A.B., Lambert, R.E., Fronczek, F.R., Cragg, P.J. & Wallace, K.J. An activated coumarin-enamine Michael acceptor for CN−. *New J. Chem.* **38**, 4678-4683 (2014).
7. Coleman, R.S. & Madaras, M.L. Synthesis of a novel coumarin C-riboside as a photophysical probe of oligonucleotide dynamics. *J. Org. Chem.* **63**, 5700-5703 (1998).
8. Wang, J., Long, L. & Xiao, X. A fast-responsive fluorescent probe for sulfite and its bioimaging. *J. Lumin.* **31**, 775-781 (2016).
9. Yang, Y., *et al.* A highly sensitive fluorescent probe for the detection of bisulfite ion and its application in living cells. *Dyes Pigm.* **136**, 830-835 (2017).
10. Anzalone, A.V., Wang, T.Y., Chen, Z. & Cornish, V.W. A Common Diaryl Ether Intermediate for the Gram-Scale Synthesis of Oxazine and Xanthene Fluorophores. *Angew. Chem. Int. Edit.* **52**, 650-654 (2013).
