# Supplementary material for: A pocket-escaping design to prevent the common interference with near-infrared fluorescent probes in vivo
Source: Nat Commun. 2020 Mar 26;11:1573. doi: 10.1038/s41467-020-15323-8 (PMC7099068; doi:10.1038/s41467-020-15323-8)
Supplement: Supplementary file 3 — Reporting Summary [file 41467_2020_15323_MOESM3_ESM.pdf]

nature research

Corresponding author(s): Chunming Wang

Last updated by author(s): Feb 20, 2020

Reporting Summary

Nature Research wishes to improve the reproducibility of the work that we publish. This form provides structure for consistency and transparency in reporting. For further information on Nature Research policies, see Authors & Referees and the Editorial Policy Checklist.

Statistics

For all statistical analyses, confirm that the following items are present in the figure legend, table legend, main text, or Methods section.

n/a	Confirmed	
☐	☒	The exact sample size (n) for each experimental group/condition, given as a discrete number and unit of measurement
☐	☒	A statement on whether measurements were taken from distinct samples or whether the same sample was measured repeatedly
☐	☒	The statistical test(s) used AND whether they are one- or two-sided<br>*Only common tests should be described solely by name; describe more complex techniques in the Methods section.*
☒	☐	A description of all covariates tested
☐	☒	A description of any assumptions or corrections, such as tests of normality and adjustment for multiple comparisons
☐	☒	A full description of the statistical parameters including central tendency (e.g. means) or other basic estimates (e.g. regression coefficient) AND variation (e.g. standard deviation) or associated estimates of uncertainty (e.g. confidence intervals)
☒	☐	For null hypothesis testing, the test statistic (e.g. F, t, r) with confidence intervals, effect sizes, degrees of freedom and P value noted<br>*Give P values as exact values whenever suitable.*
☒	☐	For Bayesian analysis, information on the choice of priors and Markov chain Monte Carlo settings
☒	☐	For hierarchical and complex designs, identification of the appropriate level for tests and full reporting of outcomes
☒	☐	Estimates of effect sizes (e.g. Cohen's d, Pearson's r), indicating how they were calculated

Our web collection on statistics for biologists contains articles on many of the points above.

Software and code

Policy information about availability of computer code

Data collection

1. 1H NMR and 13C NMR spectra were recorded on Bruker 600 MHz AVANCE III spectrometer, BRUKER Ltd.
2. Mass spectra were obtained with Thermo Scientific MSQ Plus mass spectrometer (USA).
3. Absorption spectra were collected by using HACH DR6000 UV/VIS Spectrophotometer (USA).
4. Viscosity experiments were carried out using an Ubbelohde viscometer.
5. The fluorescence imaging of probe distribution in mice and probe electrophoretic bands in native PAGE gels were respectively performed using an IVIS Lumina XR (XENOGEN, Caliper, Hopkinton, MA, USA) imaging system.
6. Column chromatography was carried out on silica gel (G200–300 mesh, Qingdao Haiyang Chemical Co., Qingdao, China).
7. Fluorescent spectra of time-course and titration experiments were recorded on FluoroMax-4 spectrofluorometer (Horiba Jobin Yvon Inc.). The other spectra were measured with a Thermo Scientific Lumina Fluorescence Spectrometer (USA).
8. Quantitative measurements of probes binding to albumin were conducted on a Malvern Microcal PEAQ ITC.
9. Haematoxylin and eosin (H&E) staining images were captured using Axio Imager A2 microscope.
10. Masson staining pictures were captured using an inverted microscope (IX73, Olympus).
11. Molecular docking of all compounds was performed via Discovery Studio (BIOVIA, USA).
12. Fluorescent probe pharmacokinetics and biodistribution were detected with FlexStation III Multi-Mode Microplate Reader (Molecular Devices, USA).

Data analysis

1. The three-dimensional structures of TICT dyes were constructed using Chem. 3D ultra 14.0 software (Chemical Structure Drawing Standard; Cambridge Soft Corporation, USA).
2. Fluorescent spectra data were plotted using Origin (Ver. 9.5) graphing software.
3. Heatmaps and histograms were drawn in GraphPad Prism software (Ver. 7.02).
4. Molecular docking of all compounds was performed via Discovery Studio Ver. 3.5 (BIOVIA, USA) as implemented through the graphical user interface CDOCKER protocol.
5. DFT calculations were carried out using the Gaussian 09 program package.
6. NMR results were analyzed using MestReNova Ver. 9.0.1 (Mnova).
7. ITC results were processed by the build-in software of Microcal PEAQ ITC analysis.

For manuscripts utilizing custom algorithms or software that are central to the research but not yet described in published literature, software must be made available to editors/reviewers. We strongly encourage code deposition in a community repository (e.g. GitHub). See the Nature Research guidelines for submitting code & software for further information.

Data

Policy information about availability of data

All manuscripts must include a data availability statement. This statement should provide the following information, where applicable:

- Accession codes, unique identifiers, or web links for publicly available datasets
- A list of figures that have associated raw data
- A description of any restrictions on data availability

The source data underlying Fig. 1D, E, 2E, F, 3E, F, 4J, Supplementary materials of Fig. S6, S7, S14, S15, and S21 are provided as a Source Data file. Protein Data Bank (http://www.rcsb.org) accession codes are 2BXC and 2BXM. The data of this study are available from the corresponding authors upon reasonable request.

Field-specific reporting

Please select the one below that is the best fit for your research. If you are not sure, read the appropriate sections before making your selection.

☒ Life sciences ☐ Behavioural & social sciences ☐ Ecological, evolutionary & environmental sciences

For a reference copy of the document with all sections, see nature.com/documents/nr-reporting-summary-flat.pdf

Life sciences study design

All studies must disclose on these points even when the disclosure is negative.

Sample size	Sample size was determined empirically for sufficient statistical power. Variations between samples were also used to determine the suitability of the sample size. The detailed information is provided in 'Source Data'.
Data exclusions	No data were excluded from analyses in the experiments.
Replication	Number of replicates have been stated in each of the panels for each figure.
Randomization	In animal studies, we randomly allocated animals into groups such that as animals were added to the experiment, the numbers of animals in each group did not significantly differ.
Blinding	We needed to investigate the difference between different groups, and such difference had not been known. Thus we did not use blinding in the study.

Reporting for specific materials, systems and methods

We require information from authors about some types of materials, experimental systems and methods used in many studies. Here, indicate whether each material, system or method listed is relevant to your study. If you are not sure if a list item applies to your research, read the appropriate section before selecting a response.

Materials & experimental systems

n/a	Involved in the study	
☒	☐	Antibodies
☐	☒	Eukaryotic cell lines
☒	☐	Palaeontology
☐	☒	Animals and other organisms
☒	☐	Human research participants
☒	☐	Clinical data

Methods

n/a	Involved in the study	
☒	☐	ChIP-seq
☒	☐	Flow cytometry
☒	☐	MRI-based neuroimaging

Eukaryotic cell lines

Policy information about cell lines

Cell line source(s)	The stellate cell line HSC-T6 and hepatocytes cell line AML 12 were both purchased from the Shanghai Cell Bank of Chinese Academy of Sciences (Shanghai, China).
Authentication	No authentication was performed.
Mycoplasma contamination	All cells were tested for mycoplasma and confirmed negative.
Commonly misidentified lines (See ICLAC register)	No commonly misidentified cell lines were used.

Animals and other organisms

Policy information about studies involving animals; ARRIVE guidelines recommended for reporting animal research

Laboratory animals	C57BL/6 mice were purchased from Vital River Laboratories (Beijing, China). Ten-week-old C57BL/6 female mice (~20 g) were fed in a specific pathogen-free (SPF) animal facility with controlled light (12 h light/dark), temperature and humidity, with food and water available.
Wild animals	This study did not involve wild animals.
Field-collected samples	No field collected samples were used in the study.
Ethics oversight	The animal protocol was approved by the Animal Care and Use Committee of Nanjing University, conformed to the Guidelines for the Care and Use of Laboratory Animals published by the National Institutes of Health, and reviewed by the Animal Ethics Committee, University of Macau. The authors have complied with all relevant animal testing and research ethical regulations.

Note that full information on the approval of the study protocol must also be provided in the manuscript.
